# Prognostic significance of tumour Ki-67 dynamics during neoadjuvant treatment in patients with breast cancer: a population-based cohort study

**DOI:** 10.1016/j.lanepe.2025.101432

**Published:** 2025-09-02

**Authors:** Maria Angeliki Toli, Xingrong Liu, Davide Massa, Stefania Lando, Caroline Boman, Nikolaos Tsiknakis, Christian Tranchell, Andri Papakonstantinou, Giuseppe Fotia, Claudio Vernieri, Valentina Guarneri, Jonas Bergh, Maria Vittoria Dieci, Louise Eriksson Bergman, Alexios Matikas, Theodoros Foukakis

**Affiliations:** a Department of Oncology and Pathology, Karolinska Institutet, Stockholm, Sweden; b Department of Surgery, Oncology and Gastroenterology (DiSCOG), University of Padova, Padova, Italy; c Unit of Biostatistics, Epidemiology and Public Health, Department of Cardiac, Thoracic, Vascular Sciences and Public Health, University of Padova, Padova, Italy; d Breast Centre, Karolinska Comprehensive Cancer Centre, Stockholm, Sweden; e Department of Oncology and Hematology-Oncology, University of Milan, Milan, Italy; f Department of Oncology and Hematology, Fondazione IRCCS Istituto Nazionale dei Tumori, Milan, Italy; g Oncology 2, Veneto Institute of Oncology IOV-IRCCS, Padova, Italy; h Department of Surgery and Oncology, Capio Sankt Göran Hospital, Stockholm, Sweden

**Keywords:** Breast cancer, Neoadjuvant chemotherapy, Ki-67, Prognosis, Survival, Stratification, Neo-Bioscore, Real-world data

## Abstract

**Background:**

Although Ki-67 is a commonly used proliferation marker in breast cancer (BC), its prognostic value after neoadjuvant chemotherapy (NACT) remains unclear. This study aims to investigate the prognostic implications of Ki-67 dynamics during NACT.

**Methods:**

Patients with invasive BC treated with NACT (2007–2020) were identified through the National Breast Cancer Register (NBCR). Associations between Ki-67 dynamics with survival outcomes were studied (spline-based Cox regression). The prognostic value of Ki-67 in the Neo-Bioscore model was examined and optimal cut-off values for relative change of Ki-67 ((post-NACT–pre-NACT Ki-67)/pre-NACT Ki-67) were explored (minimum p-value approach).

**Findings:**

Among 2494 patients, median pre-NACT Ki-67 was 40% (IQR:28–65%). Median post-NACT Ki-67 in patients with residual disease (n = 1826) was 12% (IQR:5–35%). Lower post-NACT Ki-67 was associated with better breast cancer specific survival (BCSS) in the whole cohort (p < 0.0001), in ER+/HER2− (p = 0.0001) and TNBC (p = 0.0007), but not in HER2+ BC (p = 0.8223). Post-NACT Ki-67 improved the Neo-Bioscore prognostic model increasing the C-index from 0.758 to 0.802. Post-NACT Ki-67, as well as absolute and relative change of Ki-67 were strongly correlated with each other and were prognostic for long-term outcomes. Optimal cut-off values for relative change of Ki-67 identified prognostic subgroups for ER+/HER2− BC (n = 730, p < 0.0001), and TNBC (n = 279, p < 0.0001). The results were validated in an external TNBC cohort of 221 patients (p = 0.00073). Notably, the identified low-risk patients with residual disease and at least 48% reduction of Ki-67 after NACT, had comparable survival to those with pathological complete response (pCR) (p = 0.13).

**Interpretation:**

Relative change of Ki-67 was independently prognostic for patient risk stratification. Ki-67 in residual disease warrants for further investigation when exploring post-neoadjuvant treatment strategies.

**Funding:**

10.13039/501100002794Cancerfonden, 10.13039/501100004359Vetenskapsrådet, 10.13039/501100007231Cancerföreningen i Stockholm and Region Stockholm.

**Research in context**

**Evidence before this study**

Neoadjuvant chemotherapy (NACT) plays an important role in the management of breast cancer, offering several advantages. Patients who achieve a pathological complete response (pCR) after NACT have better survival outcomes compared to those with residual disease. Thus, patients who do not achieve pCR are often considered for additional post-operative treatment according to the current international guidelines by the European Society for Medical Oncology (ESMO). To guide these treatment decisions, there is an increasing need for reliable biomarkers to better stratify patients after NACT. Ki-67 is commonly used in breast cancer as a cellular proliferation marker. We searched PubMed from inception to 07/2025 using combinations of the terms “breast cancer”, “neoadjuvant”, “Ki-67”, “cut-off”, “index”, “residual disease”, and “post-neoadjuvant”. According to the latest International Ki-67 in Breast Cancer Working Group (IKWG) consensus, identified in our search, Ki-67 cut-off values of ≤5% or ≥ 30% have validated prognostic value in estrogen receptor (ER) positive HER2 negative breast cancer, but are not established for the other subtypes. Although the prognostic role of Ki-67 is evident, the clinical significance of its dynamic changes before and after NACT and how those relate to outcomes and treatment decisions in the post-neoadjuvant setting is still evolving.

**Added value of this study**

This study examined the prognostic role of Ki-67 before and after NACT and showed that changes in Ki-67 are significantly associated with survival outcomes. In addition, Ki-67 added value to an established prognostic model for patient risk stratification after NACT. Cut-off values for Ki-67 changes after NACT that stratify patients into distinct prognostic groups were identified, including a low-risk group with residual disease and survival outcomes comparable to patients who achieved pCR.

**Implications of all the available evidence**

Changes in Ki-67 before and after NACT improve risk stratification of patients with residual disease, especially in triple-negative breast cancer. Identifying groups of patients with residual disease and excellent outcomes can help guide post-NACT treatment strategies and should be further explored in prospective studies.

## Introduction

Neoadjuvant chemotherapy (NACT) is widely utilized in the management of breast cancer (BC),^1^ demonstrating comparable efficacy to adjuvant chemotherapy.^2^ It offers several benefits,^3^ with the response to NACT and clinical outcomes being closely linked to intrinsic tumour characteristics.^4^ In particular, patients who achieve pathological complete response (pCR) following NACT have more favourable outcomes when compared with those with residual disease, regardless of specific biological tumour type i.e., triple-negative breast cancer (TNBC), vs. human epidermal growth factor receptor 2 positive (HER2+) BC vs. hormone receptor positive and HER2 negative (HR+/HER2−) BC.^3^^,^^5^^,^^6^ Most patients who do not achieve pCR during NACT are candidates for receiving intensified postoperative treatment.^1^ Identifying patients at high risk for additional treatments, or patients that respond well to NACT and require less intensive treatment after surgery,^7^ is therefore critical. With increasing options for adjuvant treatments following NACT, there is a need for reliable biomarkers and validated post-NACT prognostic scores^8^^,^^9^ to refine risk stratification and optimize treatment selection after NACT.

Ki-67 has been commonly used as a measure of cellular proliferation in BC.^10^ It is a nuclear cortex protein expressed during all cell cycle phases, except for G0 phase.^11^ Ki-67 is a known prognostic marker in early-stage BC.^10^ Especially in estrogen receptor positive (ER+) BC, validated Ki-67 cut-offs, alone or in combination with gene expression assays, are commonly used to guide treatment strategies,^1^ such as the use of adjuvant chemotherapy or the use of Cyclin-Dependent Kinase 4/6 (CDK4/6) inhibitors.^12^ In patients receiving NACT, higher baseline Ki-67 correlates with higher pCR rates,^10^^,^^13^ while high Ki-67 in residual tumour cells after NACT is associated with worse long-term outcomes.^14^^,^^15^ The predictive role of Ki-67 in the neoadjuvant endocrine treatment (NET) setting appears also to be well defined,^16^^,^^17^ where changes in Ki-67 were able to predict recurrence risk and guide adjuvant treatment. Despite the evidence of its impact, the role of Ki-67 in guiding therapy following NACT is still evolving.

In this study we aimed to investigate the prognostic implications of Ki-67 before and after NACT, evaluate the added value of Ki-67 to a currently used prognostication model for patient stratification and identify new cut-off values for Ki-67 changes during NACT.

## Methods

### Study design

This retrospective, analytical, population-based cohort study included patients diagnosed with early-stage BC and treated with NACT. The study’s objectives were to evaluate the distribution and dynamic changes of Ki-67 in primary tumours and residual disease following NACT, as well as their implications in terms of patient survival outcomes.

The study received approval by the Swedish Ethical Review Authority (2016/1303-31 with amendments 2018/1049-32, 2021-01147, and 2023-02918-02). Further informed consent from patients included in the cohort study was not required, as the collection and analysis of data from registries and patients’ records was non-interventional. Patients and tumour samples included from the Italian Validation Cohort were collected after approval from the Institutional Review Board of each Italian participating centre, IRCCS Istituto Oncologico Veneto, Padova, Italy (CESC-IOV, prot. nr. 7533–17/05/2016) and Fondazione IRCCS Istituto Nazionale Tumori, Milan, Italy (INT 92/20), in accordance with the ethical principles set in the Declaration of Helsinki. Written consent was obtained from each participant who was alive at the time of study entry. A waiver of consent was granted for deceased patients.

The study is reported according to the STROBE (Strengthening the Reporting of Observational Studies in Epidemiology) guidelines^18^ and the European Society for Medical Oncology Guidance for Reporting Oncology real-World Evidence (ESMO-GROW) checklist,^19^ available in the Supplementary material.

### Data sources and patient cohort

**Fig. 1:**
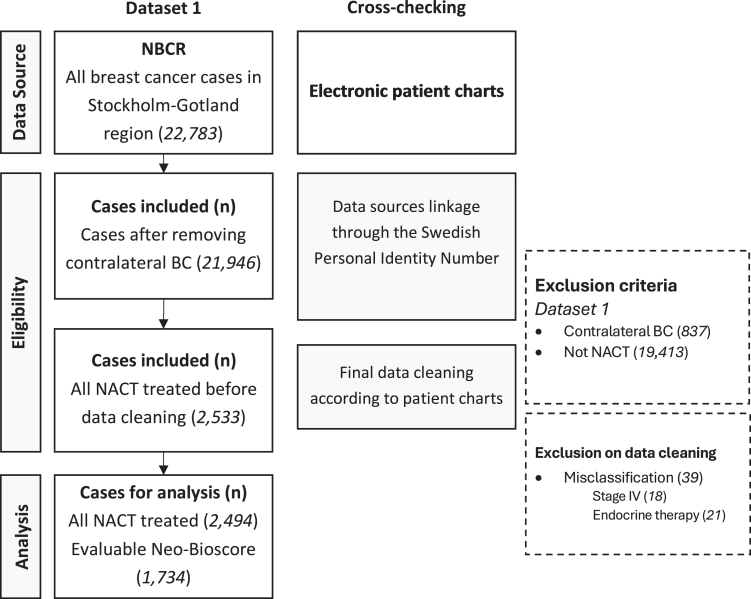
Flowchart of the study population according to the European Society for Medical Oncology Guidance for Reporting Oncology Real-World Evidence (ESMO-GROW). BC, breast cancer; NACT, neoadjuvant chemotherapy; NBCR, National Breast Cancer Register.

The patient cohort has been previously described in detail.^6^^,^^20^ All patients diagnosed with invasive BC in the Stockholm-Gotland healthcare region, which represents approximately 25% of the Swedish population, between January 1, 2007 to December 31, 2020, were identified through the National Breast Cancer Register (NBCR).^21^ Patients diagnosed with non-metastatic invasive BC, defined as having no metastatic disease within three months of breast cancer diagnosis, and treated with NACT, were included. Individuals who had contralateral BC, had no available information on NACT, received only neoadjuvant endocrine treatment, had stage IV or unknown disease stage were excluded. The detailed patient flow chart is presented in Fig. 1. The NBCR was also linked to the National Patient Register and the Cause of Death Register, to collect additional information regarding date and cause of death, as well as the site and date of distant recurrence.

The registry data were additionally complemented with information obtained from the electronic patient records. The information extracted included tumour clinicopathological characteristics like HR expression, HER2 expression and gene amplification, histological grade, Ki-67, number of removed and positive lymph nodes, tumour size, residual cancer burden (RCB) where available, and site of metastasis at baseline, residual disease, and metastasis. Details on administered neoadjuvant treatment were also collected, as well as follow-up status, including date and site of first relapse. Using the Swedish Prescribed Drug Register, information on prescribed and dispensed adjuvant capecitabine was obtained for all patients with TNBC and residual disease after NACT. No other information on adjuvant treatments was collected for the scope of this analysis. During the whole study period, patients with ER+/HER2− BC were recommended standard endocrine therapy, while CDK4/6 inhibitors were not yet approved. For HER2+ disease, one year of adjuvant trastuzumab was the standard of care throughout the study, and after 2019 adjuvant trastuzumab emtansine was used in patients with HER2+ BC and residual disease.

Additionally, pathology data were collected from the pathology reports for both the diagnostic core biopsy and the surgical specimen. The positivity threshold for ER and progesterone receptor (PR) immunohistochemistry (IHC) expression was set at ≥10%, according to Swedish national guidelines. HER2 status was defined according to 2018 guidelines from the American Society of Clinical Oncology and the College of American Pathologists (ASCO-CAP).^22^ Ki-67 was expressed as the percentage of positively stained invasive carcinoma cells.

### External validation cohort

We screened 460 patients with non-metastatic TNBC (ER and PR <10%; HER2 score of 0/1+/2+ by IHC and lack of ERBB2 gene amplification by fluorescent in situ hybridization) diagnosed between 2003 and 2023 at IRCCS Istituto Oncologico Veneto (Padova, Italy) or Fondazione IRCCS Istituto Nazionale Tumori (INT) (Milan, Italy) and treated with NACT. Of these 460 patients, 253 had residual disease after NACT; finally, 221 patients with available information on Ki-67 expression on residual disease were included in the external validation cohort. Clinicopathological, treatment, and follow-up data were collected.

### Neo-Bioscore staging system

Point assignments for the Neo-Bioscore staging system in the cohort study were defined according to the updated publication.^8^ Tumour biological characteristics contributing to the score included ER-status, histological grade, and HER2-status, and were determined on diagnostic core biopsies taken prior to NACT. Clinical stage and pathological stage were assigned according to the seventh edition of the American Joint Committee on Cancer (AJCC) staging system.^23^

### Outcomes

pCR was defined as absence of invasive tumour in the breast and the axilla, while the presence of in situ carcinoma was allowed (ypT0/Tis and ypN0). Breast cancer specific survival (BCSS) was defined as the time from primary surgery to death from breast cancer. Recurrence-free survival (RFS) was defined as the time from primary surgery to local/locoregional recurrence, distant relapse or death due to any cause, whichever occurred first. Overall survival (OS) was defined as the time from primary breast cancer surgery to death from any cause.

For individuals where follow-up was lost due to emigration, or the event of interest did not occur by the end of the study observation period (May 2, 2023), patients were censored.

### Statistical analysis

Clinical and tumour characteristics of patients included in the NACT cohort were described for the whole cohort, as well as in all patients with evaluable Neo-Bioscore. Median and interquartile range (IQR) were reported for continuous variables, while frequencies and percentages were reported for the categorical ones. Ki-67 expression was analysed as a continuous variable as recommended by the International Ki-67 in Breast Cancer Working Group.^10^ The distribution of Ki-67 expression levels pre- and post-NACT were stratified by baseline molecular tumour subtypes: ER-positive/HER2-negative, HER2-positive, and TNBC, while patients with missing data were excluded.

The association between pre-NACT Ki-67 expression and the probability to achieve pCR was illustrated according to baseline tumour subtypes. The association between Ki-67 expression and BCSS was evaluated using univariable and multivariable Cox regression analysis. Hazard ratios (HRs), comparing patients with different levels of Ki-67 to a specific reference level, were estimated and presented with 95% confidence intervals (CI). In multivariable regression analysis for pre-NACT Ki-67, Ki-67 as a continuous variable was transformed into restricted cubic splines,^24^ with adjustment for covariates such as type of chemotherapy, age at diagnosis, clinical tumour size and node status at diagnosis, ER-status, PR-status, HER2-status, histological grade, and diagnosis year. Age at diagnosis was treated as a continuous variable in all regression models. In the analysis of continuous post-NACT Ki-67 and BCSS among patients with residual disease, pre-NACT Ki-67 was added to the above multivariable model. p-values were determined based on likelihood ratio tests. For the association between pre-NACT Ki-67 and survival outcomes in the whole cohort, time zero was set as the date of breast cancer diagnosis, while for the association between post-NACT and pre-NACT Ki-67 and survival outcomes in patients with residual disease, time zero was set at the date of surgery.

The association of the Neo-Bioscore staging system with BCSS was illustrated using the Kaplan–Meier method in all patients who had evaluable Neo-Bioscore and further stratified according to tumour subtype. The Log-rank test was used to determine whether there was a statistically significant difference between these subgroups. The impact of adding Ki-67 expression to the Neo-Bioscore staging system was evaluated by fitting Cox regression models, based on the same patients with residual disease evaluable Neo-Bioscore, along with both pre- and post-NACT Ki-67 expression data. The improvement in fit was determined using the concordance index (C-index, based on 1000 times bootstrap resampling), the Akaike information criterion (AIC), and the likelihood ratio test in the multivariable analysis.

Relative change of Ki-67 expression was defined as (post-NACT Ki-67—pre-NACT Ki-67)/pre-NACT Ki-67. Absolute change of Ki-67 expression was defined as post-NACT Ki-67—pre-NACT Ki-67. Both the absolute and the relative change of Ki-67 were evaluated in association with BCSS and RFS using univariable and multivariable Cox regression. Multivariable models were adjusted for type of chemotherapy, age at diagnosis, clinical tumour size, nodal status, ER/PR/HER2 status, histological grade, and diagnosis year. In a sensitivity analysis, pre-NACT Ki-67 was also included as a covariate. Comparative analysis was performed for Ki-67 metrics and different pairs of measures of Ki-67. To ensure reliable coefficient estimates, we first screened for multicollinearity by computing Spearman correlations and variance inflation factors (VIFs) using the ‘rms' R package, for each pair of predictors (relative change of Ki-67 with pre- or post-NACT Ki-67, respectively). We then compared model fits using Harrell C-statistic (C-index) and time-dependent ROC curves (Area Under the Receiver Operating Characteristic curve, AUROC) to evaluate which Ki-67 measure or pair of measures improved prognostic performance.

Optimal cut-off values for relative change of Ki-67 were explored to stratify patients with residual disease into low- and high-risk subgroups. This was done by maximizing separation between groups based on the minimum of Log-rank test p-value and HRs (comparing lower vs. higher risk groups). Based on the grid search algorithm, we tested every candidate pair of lower and upper thresholds across the observed range, performed log-rank tests for BCSS at each split, and selected the pair yielding the smallest p-value (i.e., maximal survival separation), identifying two cut-off points produces three prognostic groups (with Ki-67 decreased, stable, and increased) without any prior assumptions about their number. Before that, unsupervised methods (Gaussian Mixture Models) were applied to investigate whether pre- or post-NACT Ki-67 predominates in defining two clusters. The identified cut-off values were subsequently evaluated through Kaplan–Meier plots. In the TNBC sub-cohort, the cut-off values were further externally validated in an independent Italian cohort of 221 patients with TNBC. In the external cohort, OS was the outcome of interest and 5-year area under curve (AUC) values were used to evaluate the performance of the cut-offs.

We performed sensitivity analysis using multiple imputations to assess robustness of results (or potential impact of missing covariates) in the association analyses between pre-NACT Ki-67 expression and BCSS, as well as post-NACT Ki-67 expression and BCSS. Missing data of covariates were imputed based on relevant covariates from the analysis, such as type of chemotherapy, patient age, clinical tumour size, node status, receptor status (ER, PR, HER2), histological grade, year of diagnosis, Ki-67 levels, and survival outcomes.

Statistical significance was defined as a two-sided p-value below 0.05. All statistical analyses and data visualization were carried out using R programming language (version 4.3.1, R Foundation for Statistical Computing, Vienna, Austria).

### Role of the funding source

The funding sources had no involvement in the design of the study, the collection, analysis, or interpretation of data, the writing of the report, or the decision to submit the manuscript for publication.

## Results

### Patient characteristics

A total of 2494 patients were included in the NACT cohort study (Fig. 1). The median age at diagnosis was 51 years. A total of 1091 (44.5%) patients had luminal BC (ER+/HER2−), 820 (33.4%) had HER2+ BC, and 543 (22.1%) had TNBC. Pre-NACT Ki-67 was missing in 61 cases and data required to calculate point assignments for Neo-Bioscore were available for 1734 patients. Baseline patient and tumour characteristics for both the entire cohort and the Neo-Bioscore cohort are shown in Table 1. The median follow-up time for BCSS was 5.87 years (IQR: 3.79–8.95).

**Table 1:** Clinical and tumour characteristics of patients with BC included in the whole NACT cohort and in patients with evaluable Neo-Bioscore.

Characteristics	Whole cohort N (%)	Neo-Bioscore cohort n (%)
**Patients, N**	2494	1734
**Age, years**		
Median (IQR)	51.6 (43.4–61.4)	51.4 (43.2–61.5)
<55	1471 (59.0)	1020 (58.8)
55 or older	1023 (41.0)	714 (41.2)
**Chemotherapy**		
Anthracycline and taxane	1995 (80.0)	1367 (78.8)
Anthracycline alone	104 (4.2)	74 (4.3)
Taxane alone	328 (13.2)	246 (14.2)
Other	67 (2.7)	47 (2.7)
**Anti-HER2 use**		
Non-user	1693 (67.9)	1168 (67.4)
User	799 (32.1)	566 (32.6)
Missing	2	
**T stage**		
cT0–2	1853 (74.5)	1280 (73.8)
cT3–4	633 (25.5)	454 (26.2)
Missing	8	
**Node status**		
Negative	1128 (45.4)	770 (44.4)
Positive	1355 (54.6)	963 (55.6)
Missing	11	1
**Grade**		
Grade 1–2	1005 (45.2)	826 (47.6)
Grade 3	1218 (54.8)	908 (52.4)
Missing	271	
**BC subtypes**		
ER+/HER2−	1091 (44.5)	793 (45.9)
HER2+	820 (33.4)	579 (33.5)
TNBC	543 (22.1)	355 (20.6)
Missing	40	7
**ER status**		
Negative	893 (35.9)	564 (32.5)
Positive	1597 (64.1)	1170 (67.5)
Missing	4	
**PR status**		
Negative	1330 (53.5)	882 (50.9)
Positive	1158 (46.5)	852 (49.1)
Missing	6	
**HER2 status**		
HER2-0	642 (27.8)	467 (26.9)
HER2-low	847 (36.7)	688 (39.7)
HER2-positive	820 (35.5)	579 (33.4)
Missing	185	
**Diagnosis year**		
2007–2010	370 (14.8)	245 (14.1)
2011–2014	538 (21.6)	376 (21.7)
2015–2018	978 (39.2)	724 (41.8)
2019–2020	608 (24.4)	389 (22.4)
**Clinical stage**		
1A	141 (5.8)	91 (5.2)
2A	946 (38.6)	656 (37.8)
2B	879 (35.9)	636 (36.7)
3A	340 (13.9)	249 (14.4)
3B	92 (3.8)	62 (3.6)
3C	53 (2.2)	40 (2.3)
Missing	43	
**Pathological stage**		
ypT0/Tis ypN0 (pCR)	661 (26.5)	301 (17.4)
1A	417 (16.7)	358 (20.6)
1B	27 (1.1)	25 (1.4)
2A	589 (23.6)	479 (27.6)
2B	263 (10.5)	218 (12.6)
3A	363 (14.6)	279 (16.1)
3C	99 (4.0)	74 (4.3)
Missing	75	
**Ki-67 pre-NACT, %**		
Median (IQR)	40.0 (28.0–65.0)	40.0 (28.0–60.0)
Missing	61	27

BC, breast cancer; NACT, neoadjuvant chemotherapy; N, number; T, tumour; ER, estrogen receptor; PR, progesterone receptor; HER2, human epidermal growth factor receptor 2; pCR, pathological complete response; IQR, interquartile range.

### Pre-NACT Ki-67 expression

**Fig. 2:**
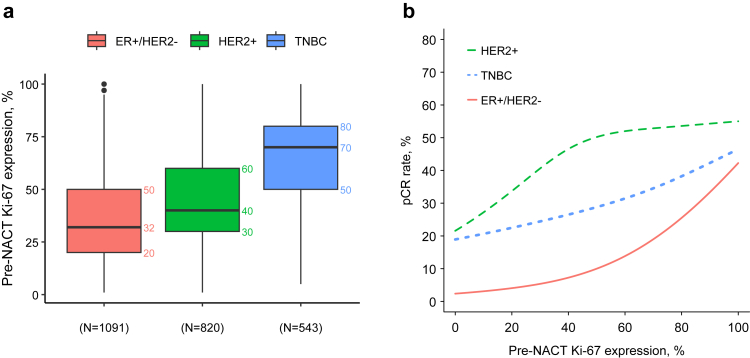
Description of (a) pre-NACT Ki-67 distributions by BC subtypes and (b) relationship of pre-NACT Ki-67 with pCR rates stratified by BC subtype. NACT, neoadjuvant chemotherapy; BC, breast cancer; pCR, pathological complete response.

Median pre-NACT Ki-67 expression, measured from the diagnostic biopsy (N = 2494), was 40% (IQR: 28–65%). TNBC tumours had the highest median Ki-67 expression of 70%, followed by HER2+ and ER+/HER2−, with 40% and 32% Ki-67 expression, respectively (Table 2). When Ki-67 expression was categorized according to the corresponding median values in each biological subgroup, we found that patients with high pre-NACT Ki-67 had higher pCR rates in ER+/HER2− BC (n = 1091, 13.1% vs. 4.4% in Ki-67 high vs. low, respectively; p < 0.0001), TNBC (n = 543, 40.1% vs. 29.2% in Ki-67 high vs. low, respectively; p = 0.0107) and HER2+ BC (n = 820, 51.7% vs. 39.7% in Ki-67 high vs. low, respectively; p = 0.0008). However, in HER2+ tumours, a plateau in pCR rates was observed after 40% of pre-NACT Ki-67 expression (Fig. 2). Additionally, when Ki-67 expression was examined in increments, pCR rates increased alongside Ki-67 levels, with the highest Ki-67 group (75% < Ki-67 ≤ 100%) showing a pCR rate of 38.7% (OR_adj_ = 3.14, 95% CI: 2.10–4.70) (Supplementary Table S1). Pre-NACT Ki-67 was found to be associated with BCSS (p = 0.022) in the whole cohort, with higher pre-NACT Ki-67 levels being linked to worse survival (Supplementary Fig. S1). However, this relationship was not linear, and for pre-NACT Ki-67 above 40%, the effect was not statistically significant. In subtype analysis, this association was not statistically significant for ER+/HER2− (p = 0.134), HER2+ (p = 0.089), or TNBC (p = 0.649) and some evidence of interaction was observed with p_int_ = 0.0504 and an interaction-term HR of 1.00 (95% CI: 0.983–1.015) for the HER2+ and 0.985 (95% CI: 0.974–0.996) for TNBC (Supplementary Fig. S1). When pre-NACT Ki-67 and RFS was examined, higher Ki-67 was associated with worse RFS (p = 0.012). However, subtype analysis showed that this association was not statistically significant for neither ER+/HER2– (p = 0.066), nor HER2+ (p = 0.435), and TNBC (p = 0.927) (Supplementary Fig. S2). The test for interaction did not reach statistical significance (p_int_ = 0.099), with interaction-term HR of 0.995 (95% CI: 0.984–1.007) for HER2+ and 0.989 (95% CI: 0.980–0.999) for TNBC. The sensitivity analysis using multiple imputations showed consistent results (Supplementary Fig. S3). When pre-NACT Ki-67 was examined in the subgroup patients with residual disease, it showed no significant association with BCSS (p = 0.238) or RFS (p = 0.066).

**Table 2:** The distribution of Ki-67 expression levels before and after NACT.

Ki-67, %	Patients (excluded missing)	Distribution measures			
		Mean (SD)	25th percentile	median	75th percentile
**Pre-NACT**	All patients	45.5 (23.5)	28	40	65
	ER+/HER2−	36.9 (21.4)	20	32	50
	HER2+	44.1 (19.2)	30	40	60
	TNBC	64.7 (22.1)	50	70	80
**Post-NACT (RD)**	Among patients with RD	24.8 (27.0)	5	12	35
	ER+/HER2− with RD	16.3 (18.7)	5	10	20
	HER2+ with RD	21.4 (22.8)	5	12	31
	TNBC with RD	50.4 (33.1)	13	55	80

NACT, neoadjuvant chemotherapy; RD, residual disease; SD, standard deviation; ER, estrogen receptor; HER2, human epidermal growth factor receptor 2; TNBC, triple negative breast cancer.

### Post-NACT Ki-67 expression

**Fig. 3:**
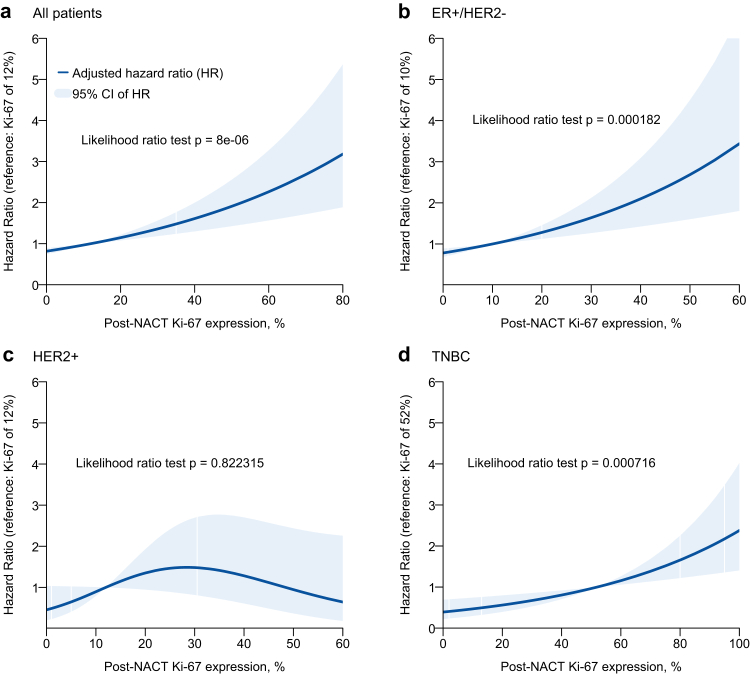
Smoothed hazard ratio plots for BCSS based on post-NACT Ki-67 with median as the reference level and adjusted for pre-NACT Ki-67, type of chemotherapy, age at diagnosis, tumour size, nodal status, ER-status, PR-status, HER2-status, grade, and year of diagnosis for (a) all 1371 patients with residual disease, and the subgroups of patients with (b) ER+/HER2− BC, (c) HER2+ BC and (d) TNBC. Ki-67 was a continuous variable and modelled as restricted cubic splines in multivariable Cox regression analyses. The plots of HR were prepared using the R function Greg::plotHR(). Time zero was the date of surgery. Adjusted HR was estimated through complete case analysis. NACT, neoadjuvant chemotherapy; ER, estrogen receptor; HER2, human epidermal growth factor receptor 2; TNBC, triple negative breast cancer; HR, hazard ratio; CI, confidence interval.

Median post-NACT Ki-67 expression, as measured in the surgical specimen of patients with residual disease (n = 1826), was 12% (IQR: 5–35%). At surgery, TBNC tumours had the highest median Ki-67 expression at 55%, followed by HER2+ tumours at 12% and ER+/HER2− tumours at 10% (detailed in Table 2). The semi-quantitative assessment using an unsupervised clustering method that incorporated pre- and post-NACT Ki-67 values identified that post-NACT Ki-67 predominates pre-NACT Ki-67 as a prognostic factor for long-term outcomes both for ER+/HER2− and TNBC subtypes (Supplementary Fig. S4). In patients with residual disease, post-NACT Ki-67 was found to be strongly prognostic for BCSS (p < 0.0001), with higher post-NACT Ki-67 levels after treatment being associated with worse survival outcomes. Sensitivity analysis further supported these results, confirming the association (Supplementary Fig. S5). After stratifying by subtype, the prognostic effect of post-NACT Ki-67 was mainly driven by ER+/HER2− (p = 0.0001) and TNBC (p = 0.0007), whereas no significant impact was seen for HER2+ (p = 0.8223) subtype (Fig. 3). A sensitivity analysis using 1% as cut-off for ER did not change the results (data not shown). A borderline statistically significant interaction between post-NACT Ki-67 and subtypes was observed with p_int_ = 0.048, and an interaction-term HR of 0.980 (95% CI: 0.962–0.998) for HER2+ and 0.986 (95% CI: 0.973–1.00) for TNBC. When post-NACT Ki-67 and RFS were examined, higher Ki-67 was strongly associated with worse RFS (p < 0.0001). Subtype analysis confirmed this effect in ER+/HER2− (p < 0.001), HER2+ (p = 0.03), and TNBC (p = 0.0021) (Supplementary Fig. S6). The interaction test was not statistically significant (p_int_ = 0.091), with interaction-term HR of 0.987 (95% CI: 0.974–1.00) for HER2+ and 0.99 (95% CI: 0.98–1.001) for TNBC.

### Independent prognostic value of Ki-67 added to Neo-Bioscore

Neo-Bioscore could be calculated in 1734 patients. The distribution of baseline characteristics of the Neo-Bioscore cohort was comparable to that of the whole cohort (Table 1) and was in line with previous studies (Supplementary Table S2).^8^^,^^9^^,^^25^ Neo-Bioscore was associated with BCSS and led to the identification of distinct prognostic groups in the whole study cohort, as well as in specific biological tumour subtypes (p < 0.001; Supplementary Fig. S7). This association was further confirmed (p < 0.001) in patients with residual disease, evaluable Neo-Bioscore, and available pre- and post-NACT Ki-67 data (n = 1085) (Supplementary Fig. S8). The prognostic performance of the Neo-Bioscore staging system was evaluated in combination with various Ki-67 measures. The addition of post-NACT Ki-67 improved the performance of the model, increasing the C-index from 0.758 to 0.802 and reducing the AIC value from 1809.78 to 1783.67 (Supplementary Fig. S9). The likelihood ratio tests were significant for all models with p < 0.001 for both ER+/HER2− and TNBC, and p = 0.027 for HER2+ subtype.

### Relative change of Ki-67

Relative change of Ki-67 was associated with BCSS (p = 0.001). Specifically, greater reductions in Ki-67’s relative change were associated with better BCSS. The statistically significant effect was also observed when stratifying by subtype (ER+/HER2−; p = 0.04, HER2+; p = 0.001, TNBC; p = 0.007) (Supplementary Fig. S10). The test for subtype interaction did not reach statistical significance (p_int_ = 0.168), with interaction-term HR of 0.83 (95% CI: 0.37–1.87) for HER2+ and 1.46 (95% CI: 0.98–2.16) for TNBC. In a sensitivity analysis adding pre-NACT Ki-67 to the relative change model, the association with BCSS remained unchanged (data not shown). Larger reductions in Ki-67 were also associated with better RFS (p < 0.001). Subtype analysis confirmed this relationship in ER+/HER2− (p = 0.004), HER2+ (p = 0.005), and TNBC (p = 0.03), and no significant interaction by subtype was detected (p_int_ = 0.734), with interaction-term HR of 0.96 (95% CI: 0.56–1.63) for HER2+ and 1.14 (95% CI: 0.81–1.61) for TNBC (Supplementary Fig. S11). The corresponding multivariable analysis on BCSS and RFS is shown in Supplementary Tables S3 and S4, respectively. Subgroup analyses performed for different calendar periods (2007–2014; p = 0.0038, 2015–2020; p = 0.0005), as well as per calendar year (p < 0.001) showed consistent results, underscoring the robustness of the findings irrespective of evolving treatment practices.

**Fig. 4:**
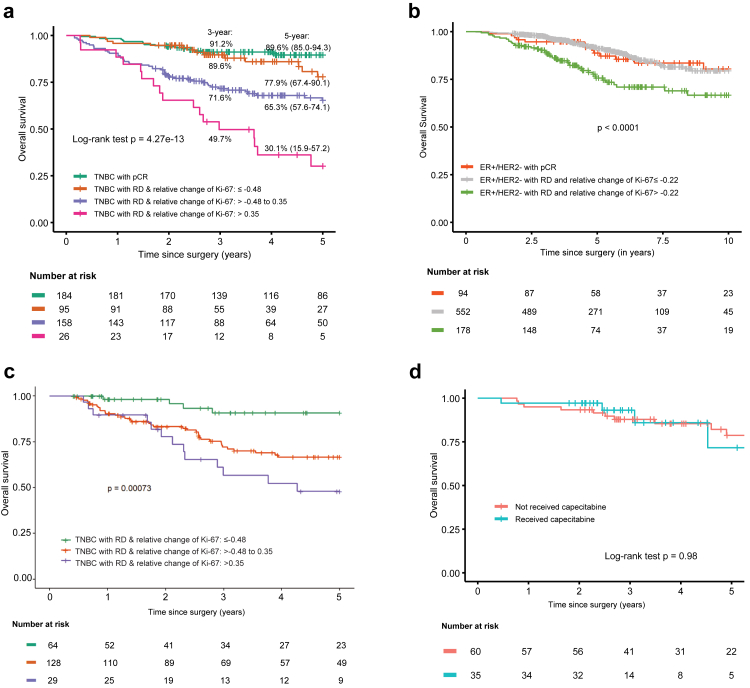
Kaplan–Meier curves of overall survival (a) in patients with TNBC and pCR (n = 184) or residual disease (RD) (n = 279) classified in three groups based on the relative change of Ki-67, (b) in patients with ER+/HER2− BC and pCR (n = 94) or residual disease (n = 730) classified in two groups based on the relative change of Ki-67, (c) in the independent validation cohort of patients with TNBC (n = 221) stratified in three groups based on the relative change of Ki-67, and (d) with or without adjuvant capecitabine in patients with TNBC and residual disease classified as low risk based on the relative change of Ki-67 (n = 95). TNBC, Triple negative breast cancer; pCR, pathological complete response; RD, residual disease; ER, estrogen receptor; HER2, human epidermal growth factor receptor 2; BC, breast cancer.

The relative change of Ki-67 expression post- vs. pre-NACT, as well as the optimal cut-off values were further examined in patients with residual disease across the specific biological tumour subtypes. In the cohort of patients with ER+/HER2− BC and with available pre- and post-NACT Ki-67 expression data (n = 730), dual optimal cut-off values for relative change of Ki-67 were identified at −0.22 and 2.85. However, only six patients had a relative change of Ki-67 greater than 2.85. Patients with relative change equal or less than −0.22, i.e., with at least 22% reduction in Ki-67 after NACT, had significantly better prognosis (p < 0.0001; Fig. 4) and comparable outcomes to those of patients with pCR (p < 0.0001; Fig. 4). Clinical and tumour characteristics of patients in each risk group are shown in Supplementary Table S5.

In patients with HER2+ BC and residual disease, no optimal cut-off values for relative change of Ki-67 expression were identified based on the available data, indicating the lack of prognostic value for Ki-67 expression dynamics in this biological setting.

In patients with TNBC (n = 279), the dual optimal cut-off values for relative change of Ki-67 expression were identified at −0.48 (i.e., a 48% Ki-67 reduction in post- vs. pre-NACT specimens) and 0.35 (i.e., a 35% Ki-67 increase in post- vs. pre-NACT specimens), defining three groups with distinct prognosis (p < 0.0001). Patient and tumour characteristics by risk group are detailed in Supplementary Table S5. Notably, the low-risk group with residual disease and relative change equal or less than −0.48, i.e., with at least a 48% reduction in Ki-67 after NACT, had comparable OS to patients with pCR (p = 0.13; Fig. 4), however the 5-year OS was numerically worse 77.9% (95% CI: 67.4–90.1), vs. 89.6% (95% CI: 85.0–94.3) in the pCR group. The prognostic value of the relative change of Ki-67 was independent from adjuvant capecitabine treatment with no significant differences in OS observed between patients who did or did not receive capecitabine (Supplementary Tables S6 and S7, Fig. 4). To validate the identified cut-offs, an external cohort of 221 patients with TNBC was used. Baseline patient and tumour characteristics for this cohort are presented in Supplementary Table S8. The external validation confirmed the prognostic significance of the dual cut-offs (p = 0.00073, 5-year AUC = 0.792, Fig. 4).

### Comparison between Ki-67 metrics

As both the post-NACT Ki-67 and the relative change of Ki-67 were significantly associated with long-term outcomes, we aimed to compare them and other Ki-67 derived metrics in an effort to identify the one that is most informative in risk stratification of patients treated with NACT. First, we checked for potential multicollinearity between relative change of Ki-67 and pre- or post-NACT Ki-67. Spearman correlations revealed almost no relationship between relative change and pre-NACT Ki-67 (r = −0.06) but showed a strong positive correlation between relative change and post-NACT Ki-67 (r = 0.85), as well as between relative and absolute change (r = 0.88) (detailed heatmap shown in Supplementary Fig. S12). Additionally, by using variance inflation factors (VIFs), we confirmed multicollinearity in the regression model that included relative change of Ki-67 and post-NACT Ki-67 (VIF >5), thus, limiting interpretability of individual regression coefficients.

Then, univariable and multivariable Cox regression model fits for all metrics and their combinations were compared using C-index (Harell C-statistic) and time-dependent ROC curve analysis (with the corresponding area under ROC curves, AUROC). This exploratory analysis indicated that when used as a continuous variable, the different Ki-67 metrics had comparable prognostic value with no significant differences between them (Supplementary Table S9).

However, when we compared the performance of Ki-67 dynamics vs. post-NACT Ki-67 for classifying patients with TNBC and residual disease, using optimal cut-off values defined with the same methodology, both crude and adjusted HRs were clearly superior in the models of the change of Ki-67 (Supplementary Table S10), proving its advantage when used for risk stratification. As expected, no significant differences were seen between the relative and absolute change of Ki-67.

## Discussion

In this retrospective, population-based cohort study, we confirmed that high pre-NACT Ki-67 expression was associated with pCR rates after NACT, while high post-NACT Ki-67 expression was associated with worse prognosis in patients with residual disease. More importantly, we showed that Ki-67 modulation during NACT has remarkable prognostic value in patients with ER+HER2− BC and TNBC failing to achieve pCR, and we identified and validated cut-off values for relative change of Ki-67 expression as an alternative approach to stratify patients after NACT.

High Ki-67 expression before NACT is a known prognostic factor associated with higher pCR rates, which we confirm in this study.^10^^,^^13^ However, the value of measuring Ki-67 during treatment or in the post-neoadjuvant setting is less explored, an exception being Ki-67 in residual disease after NET in ER+/HER2− disease. Indeed, the ADAPT and P024 trials showed that on-treatment Ki-67 levels had greater prognostic ability compared to baseline Ki-67 levels.^26^^,^^27^ Regarding the prognostic role of Ki-67 expression in residual tumour cells after NACT, previous studies indicated that Ki-67 values have prognostic role in patients with ER+/HER2− and TNBC subtypes.^14^^,^^15^ Results of our study confirm these observations in a large, contemporary, population-based cohort. However, in HER2+ tumours, the use of modern HER2-targeted therapy seems to attenuate the prognostic value of Ki-67. To further demonstrate the independent prognostic information of post-NACT Ki-67 expression, we showed that it adds significant value to the Neo-Bioscore staging system.

The most novel and clinically interesting result of our study is the finding that Ki-67 expression modulation during NACT is significantly associated with long-term clinical outcomes. The prognostic significance of measuring Ki-67 dynamics during treatment has been demonstrated in patients treated with NET in the POETIC trial, showing that persistently high levels of Ki-67 after 2 weeks of NET were associated with worse prognosis.^17^ Here, we propose the evaluation of relative change of Ki-67 expression as a more sensitive and informative measure of response to chemotherapy and long-term outcomes. We demonstrated that there is a highly significant prognostic association between relative change of Ki-67 and the examined survival outcomes across the whole cohort and per subtype analysis including HER2+ BC. In comparison to static pre- and post-NACT Ki-67, the relative change captures the tumour response to NACT and seems to be especially valuable in HER2+ subtype where absolute post-NACT Ki-67 values may be more heterogeneous due to targeted anti-HER2 therapy. Additionally, we defined and validated cut-off values for relative change of Ki-67 in patients with residual disease and identified a low-risk group of patients in both ER+/HER- and TNBC, who have an excellent prognosis, comparable to that of patients with pCR. These results confirm that the relative change of Ki-67, as an indicator of biological response, could capture treatment-induced changes that static biomarkers might miss.

Currently, patients with HER2+ BC, or TNBC and residual disease after NACT are treated with additional adjuvant therapies, such as trastuzumab emtansine (T-DM1) in the case of HER2+ BC and capecitabine in patients with TNBC, with novel experimental antibody-drug conjugates (ADCs) being currently evaluated; however, only a relatively small subset of these patients will undergo tumour relapse, thus exposing the majority of them to potentially toxic treatments.^28^^,^^29^ Therefore, better prognostic models should be developed to identify HER2+ BC and TNBC patients who actually need medical treatment escalation after failing to achieve pCR during NACT. Similar needs exist in patients with high-risk ER+/HER2− tumours, who are currently offered extended adjuvant endocrine therapy plus CDK4/6 inhibitors, with no possibility of de-escalation in patients with favourable prognosis based on their response to NACT. Therefore, the identification of a group with favourable prognosis, despite the presence of residual disease following NACT may, upon further validation, lead to a safe de-escalation of adjuvant treatment without compromising long-term outcomes.

The population-based cohort and size of our study aimed to reduce selection bias; however, there are some limitations that need to be acknowledged. Missingness and misclassification from the registry decreased after cross-checking with the electronic patient records, yet tumour grade was still inadequately reported as it was not consistently analysed routinely in pretreatment biopsies. To mitigate this bias, multiple imputations were used to handle missing data and to ensure robustness of results. Due to missing data, the Neo-Bioscore staging system could not be calculated for 760 individuals. We could however show that the remaining 1734 patients were representative of the whole cohort, both in terms of clinicopathological characteristics and in the distribution of Neo-Bioscore groups. Another limitation of our study is the lack of RCB score, which is used to assess response to NACT and has prognostic value in TNBC. The identified cut-off values for ER+/HER2− BC were not validated in an external cohort, unlike TNBC, which may limit generalizability. It is also important to note that the NACT regimens and indications as well as post-neoadjuvant therapies have evolved during the years of the study. The multivariable association analyses were however adjusted for the year of diagnosis, and the results were robust throughout the study period. Lastly, pre-analytical and analytical factors could not be controlled, but as reported by other studies, despite the differences in antibodies, detection systems and protocols across labs in Sweden, there were no specific factors causing inter- or intra-laboratory variability.^30^ In the current study, the inherent method-dependent limitations are, however, mitigated by investigating the relative changes of Ki-67 as for the individual patients, the analysis was generally performed in the same laboratory using the same protocol and scoring method. For the same reason, although our study could not directly distinguish which of the Ki-67 metrics is better, the generalisability of the relative change of Ki-67 (being a ratio) is deemed higher and was thus selected for external validation and further development.

In conclusion, our study confirmed that post-NACT Ki-67 is a significant prognostic marker in ER+/HER2− BC and TNBC, but not in HER2+ BC. Patients with residual disease and a relative reduction of Ki-67 of more than 20% in ER+/HER2− BC or more than 50% in TNBC have excellent prognosis, which is comparable to the prognosis of patients achieving pCR during NACT. These findings warrant further assessment of the relative change of Ki-67 expression to guide post-NACT treatment strategies.

## Contributors

Concept and design: MAT, LEB, AM, TF. Data acquisition and verification: MAT, CB, CT, LEB, AM, DM, VG, MVD, GF. Statistical analysis and access to raw data: XL, SL, NT. Interpretation: MAT, XL, JB, AP, CV, LEB, AM, TF. Original draft: MAT, XL. Review and editing: All authors. MAT and TF had final responsibility for the decision to submit the manuscript for publication.

## Data sharing statement

The dataset used in this study is managed by the National Breast Cancer Register (NBCR) and may be made available upon request, subject to appropriate approvals. Access to data from the independent validation cohort requires prior approval from the relevant Ethics Committee and the establishment of a Data Transfer Agreement. Interested researchers may contact the Department of Surgery, Oncology and Gastroenterology at the University of Padua (ricerca.discog@unipd.it).

## Declaration of interests

The authors report the following, all outside the submitted work. DM: travel Expenses from Eli Lilly, Pfizer. AP: research grant Pfizer (institutional payment). CV: advisory role for Eli Lilly, Novartis, Pfizer, Menarini Stemline, Daiichi Sankyo, AstraZeneca; consultancy activity for Eli Lilly and Novartis; speaker honoraria from Eli Lilly, Menarini Stemline, MSD, Novartis, Pfizer, AstraZeneca, Istituto Gentili, Accademia Nazionale di Medicina; research funding (to the institution) from Roche. VG: personal fees for advisory board membership for AstraZeneca, Daiichi Sankyo, Eli Lilly, Gilead, MSD, Novartis, Pfizer, Olema Oncology, Pierre Fabre, Roche, Menarini Stemline; personal fees as an invited speaker for AstraZeneca, Daiichi Sankyo, Eli Lilly, Exact Sciences, Gilead, GSK, Novartis, Roche, Zentiva, Menarini Stemline; personal fees for expert testimony for Eli Lilly; patent: listed as co-inventor on patent application of HER2-DX. JB: Research grants from Amgen, AstraZeneca, Bayer, Merck, Pfizer, Roche and Sanofi-Aventis to Karolinska Institutet and/or University Hospital (no personal payments); Honoraria from Roche and AstraZeneca for chairmanship and lectures at scientific meetings; consultations for Novartis, for Stratipath AB; payments to Coronis and Asklepios Cancer Research AB; stocks in Stratipath AB (the company is involved in AI based diagnostics for breast cancer); co-author on two chapters in UpToDate: 1) Deciding when to use adjuvant chemotherapy for hormone receptor-positive, HER2-negative breast cancer. 2) Prognostic and predictive factors in early, non-metastatic breast cancer; honoraria to Asklepios Medicin HB; honoraria from Roche and AstraZeneca for chairmanship and lectures at scientific meetings, patient/“case discussions” at a postgraduate course; consultations and lectures for Novartis, for Stratipath AB; payments to Coronis and Asklepios Cancer Research AB. MVD: consultancy/advisory role for Novartis, Eli Lilly, Seagen, Exact Science, Pfizer, Daiichi Sankyo, Gilead, MSD, AstraZeneca; invited speaker for Exact Sciences, Daiichi Sankyo, Novartis, Roche, Eli Lilly, AstraZeneca; research grant from Roche (Institutional); travel expenses from Eli Lilly, Roche, Gilead, Daiichi Sankyo; patent: listed as co-inventor on patent application of HER2-DX. AM: principal investigator for clinical trials sponsored by AstraZeneca and MSD; consultant for Veracyte, Roche, Seagen, and AstraZeneca; institutional grant/research support from MSD, AstraZeneca, Novartis, and Veracyte. TF: invited Speaker, Payment to Institution: Roche, Astra Zeneca, Gilead Sciences, Novartis, Daiichi Sankyo; royalties, personal: Wolters Kluwer (authorship of two chapters in UpToDate); clinical trial support to Institution: Astra Zeneca, Coordinating PI (research grant and study drug), Novartis, Coordinating PI (research grant and study drug), Veracyte, Coordinating PI (discount on the ProsignaPAM50 assay in ARIADNE clinical trial). The remaining authors have no conflicts of interest to report.

## Appendix A Supplementary data

Supplementary Figs. S1–S12 and Tables S1–S10

ESMO-GROW checklist
