# Supplementary material for: Prognostic significance of tumour Ki-67 dynamics during neoadjuvant treatment in patients with breast cancer: a population-based cohort study
Source: Lancet Reg Health Eur. 2025 Sep 2;58:101432. doi: 10.1016/j.lanepe.2025.101432 (PMC12444493; doi:10.1016/j.lanepe.2025.101432)
Supplement: Supplementary Figs. S1–S12 and Tables S1–S10 [file mmc1.pdf]

Title: Prognostic significance of tumour Ki-67 dynamics during neoadjuvant treatment in patients with breast cancer: a population-based cohort study

Authorship: Maria Angeliki Toli, MD[1]; Xingrong Liu, PhD[1]; Davide Massa, MD[2]; Stefania Lando, MSc[2,3]; Caroline Boman, MD[1,4]; Nikolaos Tsiknakis, MEng[1]; Christian Tranchell, MD[4]; Andri Papakonstantinou, MD[1,4]; Giuseppe Fotia, MD[5,6]; Claudio Vernieri, MD[5,6]; Valentina Guarneri, MD[2,7]; Jonas Bergh, MD[1,4]; Maria Vittoria Dieci, MD[2,7]; Louise Eriksson Bergman, MD[1,8]; Alexios Matikas, MD[1,4]; Theodoros Foukakis, MD[1,4]

[1]Department of Oncology and Pathology, Karolinska Institutet, Stockholm, Sweden

[2]Department of Surgery, Oncology and Gastroenterology (DiSCOG), University of Padova, Padova, Italy

[3]Unit of Biostatistics, Epidemiology and Public Health, Department of Cardiac, Thoracic, Vascular Sciences and Public Health, University of Padova, Padova, Italy

[4]Breast Centre, Karolinska Comprehensive Cancer Centre, Stockholm, Sweden

[5]Department of Oncology and Hematology-Oncology, University of Milan, Milan, Italy

[6]Department of Oncology and Hematology; Fondazione IRCCS Istituto Nazionale dei Tumori, Milan, Italy

[7]Oncology 2, Veneto Institute of Oncology IOV-IRCCS, Padova, Italy

[8]Department of Surgery and Oncology, Capio Sankt Göran Hospital, Stockholm, Sweden

Corresponding author: Maria Angeliki Toli, MD

Department of Oncology and Pathology, Karolinska Institutet, 17164 Stockholm, Sweden

Email: maria.angeliki.toli@ki.se, Tel: +46 704122116

Table of Contents

Table S1. Association of pCR with increment in Ki-67 proliferation by ORadj 3

Table S2. Distribution of patients in each group identified by Neo-Bioscore 3

Table S3. Multivariable analysis on breast cancer specific survival (BCSS) 4

Table S4. Multivariable analysis on recurrence-free survival (RFS) 5

Table S5. Clinical and tumour characteristics of patients in each subgroup identified by optimal cut-off values of relative change of Ki-67. 6

Table S6. Distribution of adjuvant capecitabine across calendar years 7

Table S7. Distribution of adjuvant capecitabine across TNBC risk-groups by optimal cut-offs of relative change of Ki-67 7

Table S8. Clinical and tumour characteristics of the independent Italian cohort of patients with TNBC 8

Table S9. Comparison of Ki-67 metrics and different pairs of measures of Ki-67....................9

Table S10. Crude and adjusted HRs for TNBC risk stratification (using cut-offs) by relative change of Ki-67 vs post-NACT Ki-67 vs absolute change of Ki-67....................10

Figure S1. Association between pre-NACT Ki-67 and BCSS....................11

Figure S2. Association between pre-NACT Ki-67 and RFS 12

Figure S3. Sensitivity analysis for missing covariates for pre-NACT Ki-67 13

Figure S4. Unsupervised clustering of patients with residual disease using pre- and post-NACT Ki-67 14

Figure S5. Sensitivity analysis for missing covariates for post-NACT Ki-67 15

Figure S6. Association between post-NACT Ki-67 and RFS....................16

Figure S7. Association between Neo-Bioscore (among patients with evaluable) and BCSS 17

Figure S8. Association between Neo-Bioscore (among patients with evaluable Neo-Bioscore, pre- and post-NACT Ki-67) and BCSS 18

Figure S9. Prognostic performance of Neo-Bioscore with the addition of Ki-67 19

Figure S10. Association between relative change of Ki-67 and BCSS 20

Figure S11. Association between relative change of Ki-67 and RFS 21

Figure S12. Spearman correlation heatmap for different Ki-67 metrics 22

References 22

Table S1. Association of pathological complete response (pCR) with increment in Ki-67 proliferation supported by adjusted odds ratio (ORadj), as well as overall and stratified pCR rates by BC subtype.

**Category (by % interval)**	**Overall**		**Multivariable Analysis***	**pCR rates by subtype, %**		
	**N (%)**	**pCR rate, %**	**OR_{adj} (95% CI)**	**HER2+**	**TNBC**	**ER+/HER2-**
1 ≤ Ki-67 ≤ 25	563 (23·1)	11·8	1·0 [reference]	32·4	17·1	4·2
25 < Ki-67 ≤ 50	1020 (41·9)	24·9	1·57 (1·13-2·19)	44·4	28·7	6·2
50 < Ki-67 ≤ 75	498 (20·5)	37·8	2·49 (1·74-3·57)	55·4	32·9	17·5
75 < Ki-67 ≤ 100	352 (14·5)	38·7	3·14 (2·10-4·70)	49·1	40·6	28·2

*Multivariable analysis included covariates such as chemotherapy, T stage, node status, ER status, PR status, HER2 status, age at diagnosis, and diagnosis year.
Abbreviations: pCR, pathological complete response; ER, Estrogen receptor; HER2, Human epidermal growth factor receptor 2; TNBC, Triple negative breast cancer.

Table S2. Descriptive statistics of 1734 patients with evaluable Neo-Bioscore across subgroups stratified by Neo-Bioscore staging system, comparing the current cohort with published data in literature.

**Neo-Bioscore**	**Current cohort n (%)**	**Published cohort data in literature,[1-3] n (%)**		
		**Study 1**	**Study 2**	**Study 3**
0	43 (2·5)	32 (1·3)	5 (0·7)	14 (1·7)
1	201 (11·6)	187 (7·9)	54 (7·2)	56 (7·0)
2	452 (26·1)	520 (21·9)	147 (19·6)	171 (21·3)
3	587 (33·9)	776 (32·6)	235 (31·3)	293 (36·5)
4	316 (18·2)	517 (21·8)	227 (30·3)	175 (21·8)
5	104 (6·0)	265 (11·1)	76 (10·1)	77 (9·6)
6	27 (1·6)	71 (3·0)	6 (0·8)	13 (1·6)
7	4 (0·2)	9 (0·4)	0 (0)	3 (0·4)
Total N	1734	2377	750	802

Study 1 (the original study): Mittendorf, E. A., Vila, J., Tucker, S. L., Chavez-MacGregor, M., Smith, B. D., Symmans, W. F., ... & Hunt, K. K. (2016). The neo-bioscore update for staging breast cancer treated with neoadjuvant chemotherapy: incorporation of prognostic biologic factors into staging after treatment. JAMA oncology, 2(7), 929-936.
Study 2: Laas, E., Labrosse, J., Hamy, A. S., Benchimol, G., de Croze, D., Feron, J. G., ... & Reyal, F. (2021). Determination of breast cancer prognosis after neoadjuvant chemotherapy: comparison of Residual Cancer Burden (RCB) and Neo-Bioscore. British Journal of Cancer, 124(8), 1421-1427.
Study 3: Kantor, O., Laws, A., Pastorello, R. G., King, C., Wong, S., Dey, T., ... & Mittendorf, E. A. (2021). Comparison of breast cancer staging systems after neoadjuvant chemotherapy. Annals of surgical oncology, 28(12), 7347-7355.

Table S3. Results of multivariable analysis on breast cancer specific survival (BCSS), mainly presented with hazard ratios of covariates based on all corresponding patients.

	**Hazard ratios* (95% CI)**		
**Exposure of interest**	**Pre-NACT Ki-67**	**Post-NACT Ki-67**	**Relative change of Ki-67**
**Pre-NACT Ki-67**	-	0·997 (0·988-1·01)	-
**Age, years**	1·02 (1·01-1·03)	1·02 (1·01-1·03)	1·02 (1·00-1·03)
**Chemotherapy**			
Anthracycline and Taxane	1·00 (reference)	1·00 (reference)	1·00 (reference)
Anthracycline	0·92 (0·58-1·45)	0·72 (0·39-1·31)	0·74 (0·41-1·35)
Taxane	0·46 (0·25-0·83)	0·46 (0·23-0·91)	0·47 (0·24-0·94)
Other	0·69 (0·21-2·22)	0·66 (0·16-2·78)	0·59 (0·14-2·49)
**T stage**			
T0-2	1·00 (reference)	1·00 (reference)	1·00 (reference)
T3-4	1·12 (0·87-1·45)	1·23 (0·87-1·75)	1·25 (0·88-1·77)
**Nodal status**			
Negative	1·00 (reference)	1·00 (reference)	1·00 (reference)
Positive	1·71 (1·32-2·21)	2·12 (1·49-3·01)	2·04 (1·44-2·89)
**Grade**			
Grade 1-2	1·00 (reference)	1·00 (reference)	1·00 (reference)
Grade 3	1·01 (0·75-1·35)	1·01 (0·67-1·53)	1·26 (0·87-1·83)
**Estrogen receptor**			
Positive	1·00 (reference)	1·00 (reference)	1·00 (reference)
Negative	1·54 (1·11-2·14)	1·56 (0·96-2·55)	1·89 (1·19-3·02)
**Progesterone receptor**			
Positive	1·00 (reference)	1·00 (reference)	1·00 (reference)
Negative	1·30 (0·94-1·79)	1·40 (0·89-2·21)	1·52 (0·97-2·39)
**HER2 status**			
Positive	1·00 (reference)	1·00 (reference)	1·00 (reference)
Negative	2·85 (2·06-3·93)	1·90 (1·19-3·02)	1·99 (1·25-3·16)
**Diagnosis year**			
2007-2010	1·00 (reference)	1·00 (reference)	1·00 (reference)
2011-2014	0·83 (0·61-1·12)	0·74 (0·45-1·21)	0·72 (0·44-1·17)
2015-2018	0·43 (0·30-0·60)	0·52 (0·31-0·88)	0·51 (0·31-0·85)
2019-2020	0·26 (0·14-0·45)	0·31 (0·15-0·64)	0·30 (0·14-0·61)

*All HRs and 95% CIs were estimated based on complete case analyses.
Abbreviations: CI, Confidence interval; NACT, neoadjuvant chemotherapy; T, tumour; HER2, human epidermal growth factor receptor 2.

Table S4. Results of multivariable analysis on recurrence-free survival (RFS), mainly presented with hazard ratios of covariates based on all corresponding patients.

	**Hazard ratios* (95% CI)**		
**Exposure of interest**	**Pre-NACT Ki-67**	**Post-NACT Ki-67**	**Relative change of Ki-67**
**Pre-NACT Ki-67**	-	1·00 (0·99-1·01)	-
**Age, years**	1·01 (1·00-1·02)	1·01 (1·00-1·02)	1·01 (1·00-1·02)
**Chemotherapy**			
Anthracycline and Taxane	1·00 (reference)	1·00 (reference)	1·00 (reference)
Anthracycline	1·14 (0·78-1·66)	0·87 (0·54-1·42)	0·92 (0·57-1·49)
Taxane	0·90 (0·64-1·28)	0·75 (0·50-1·14)	0·76 (0·51-1·15)
Other	1·01 (0·46-2·18)	0·96 (0·38-2·42)	0·89 (0·36-2·24)
**T stage**			
T0-2	1·00 (reference)	1·00 (reference)	1·00 (reference)
T3-4	1·28 (1·04-1·58)	1·34 (1·03-1·75)	1·34 (1·03-1·76)
**Node status**			
Negative	1·00 (reference)	1·00 (reference)	1·00 (reference)
Positive	1·54 (1·26-1·89)	1·79 (1·38-2·33)	1·75 (1·35-2·28)
**Grade**			
Grade 1-2	1·00 (reference)	1·00 (reference)	1·00 (reference)
Grade 3	0·97 (0·76-1·22)	0·98 (0·72-1·33)	1·20 (0·91-1·59)
**Estrogen receptor**			
Positive	1·00 (reference)	1·00 (reference)	1·00 (reference)
Negative	1·22 (0·93-1·59)	1·19 (0·82-1·70)	1·42 (1·01-2·01)
**Progesterone receptor**			
Positive	1·00 (reference)	1·00 (reference)	1·00 (reference)
Negative	1·36 (1·05-1·76)	1·55 (1·11-2·17)	1·69 (1·21-2·35)
**HER2 status**			
Positive	1·00 (reference)	1·00 (reference)	1·00 (reference)
Negative	2·16 (1·69-2·76)	1·44 (1·04-1·98)	1·48 (1·07-2·04)
**Diagnosis year**			
2007-2010	1·00 (reference)	1·00 (reference)	1·00 (reference)
2011-2014	0·98 (0·75-1·28)	0·99 (0·65-1·52)	0·98 (0·64-1·50)
2015-2018	0·53 (0·39-0·70)	0·66 (0·43-1·03)	0·66 (0·43-1·03)
2019-2020	0·43 (0·29-0·63)	0·58 (0·35-0·97)	0·58 (0·35-0·96)

*All HRs and 95% CIs were estimated based on complete case analyses.
Abbreviations: CI, Confidence interval; NACT, neoadjuvant chemotherapy; T, tumour; HER2, human epidermal growth factor receptor 2.

Table S5. Clinical and tumour characteristics of the subset of patients with residual disease (RD) stratified into risk groups by optimal cut-off values of relative change of Ki-67.

	**Patients with TNBC and RD (n=279)**		**Patients with ER+HER2- and RD (n=730)**	
	**Lower risk group ***	**Higher risk group**	**Lower risk group ****	**Higher risk group**
**No. of patients**	95	184	552	178
**Diagnosis age, years**				
< 55	56 (59%)	113 (61%)	329 (60%)	81 (46%)
≥ 55	39 (41%)	71 (39%)	223 (40%)	97 (54%)
**Clinical stage**				
I/IIA	62 (67%)	84 (46%)	224 (41%)	70 (40%)
IIB/IIIA	25 (27%)	87 (48%)	293 (54%)	93 (53%)
IIIB/IIIC	5 (5%)	10 (6%)	27 (5%)	11 (6%)
Missing	3	3	8	4
**Clinical nodes**				
Negative	63 (68%)	88 (48%)	235 (43%)	67 (38%)
Positive	30 (32%)	94 (52%)	316 (57%)	110 (62%)
Missing	2	2	1	1
**Pathological Stage**				
0/I	60 (65%)	64 (36%)	87 (17%)	27 (16%)
IIA/IIB/IIIA/IIIB	31 (34%)	98 (56%)	396 (77%)	125 (75%)
IIIC	1 (1%)	14 (8%)	34 (7%)	15 (9%)
Missing	3	8	35	11
**Pathologic nodes**				
Negative	68 (73%)	88(48%)	147 (27%)	48 (28%)
Positive	25 (27%)	94 (52%)	392 (73%)	125 (72%)
Missing	2	2	13	5
**Pathological grade**				
Grade 1	3 (4%)	0	77 (16%)	18 (11%)
Grade 2	63 (74%)	27 (17%)	360 (75%)	72 (44%)
Grade 3	19 (22%)	134 (83%)	40 (8%)	73 (45%)

Missing	10	23	75	15

* For patients with TNBC, the lower risk group was defined for those who had relative change of Ki-67 ≤ -0.48, otherwise > -0.48 in the higher-risk group.
** For patients with ER+HER2-, the lower risk group was defined for those who had relative change of Ki-67 ≤ -0.22, otherwise > -0.22 in the higher-risk group.
Abbreviations: ER, Estrogen receptor; HER2, Human epidermal growth factor receptor 2; TNBC, Triple negative breast cancer; RD, residual disease.

Table S6. Distribution of adjuvant capecitabine across calendar years for all 357 patients with triple negative breast cancer (TNBC) and residual disease (RD).

Calendar period	Number of patients, n	Adjuvant capecitabine	
		Not received, n (%)	Received, n (%)
2007-2010	34	34 (100)	0
2011-2014	77	61 (79·2)	16 (20·8)
2015-2018	126	78 (61·9)	48 (38·1)
2019-2020	120	51 (42·5)	69 (57·5)

Table S7. Distribution of adjuvant capecitabine across risk-groups by optimal cut-offs of relative change of Ki-67 for 279 patients with triple negative breast cancer (TNBC), residual disease (RD) and available pre- and post-NACT (neoadjuvant) Ki-67.

3-Categories (by relative change of Ki-67)	Number of patients in each risk group	Adjuvant capecitabine	
		Not received, n (%)	Received, n (%)
Low-risk group (≤ - 0·48)	95	60 (63·2)	35 (36·8)
Intermediate-risk group (> - 0·48 to 0·35)	158	82 (51·9)	76 (48·1)
High-risk group (> 0·35)	26	16 (61·5)	10 (38·5)

Table S8. Clinical and tumour characteristics of the independent Italian cohort of patients with triple negative breast cancer (TNBC), used for the external validation of the optimal cut-off values for post-NACT Ki-67 and relative change (RC) of Ki-67.

**Characteristics**	**Italian cohort**<br>**N (%)**
**Patients, N**	221
**Age, years**	
median (IQR)	52 (43-60)
Mean (SD)	52·7 (13)
**Chemotherapy**	
Anthracycline and taxane	202 (91·4)
Anthracycline alone	3 (1·4)
Taxane alone	15 (6·8)
Other	1 (0·5)
**T stage**	
cT0-2	151 (70·6)
cT3-4	63 (29·4)
Missing	7
**Node status**	
Negative	108 (49·8)
Positive	109 (50·2)
Missing	4
**Grade**	
Grade 2	22 (10·2)
Grade 3	194 (89·8)
Missing	5
**Diagnosis year**	
2003-2009	17 (7·7)
2010-2016	85 (38·5)
2017-2023	119 (53·8)
2019-2020	68 (24·4)
**Stage**	
1	21 (9·5)
2	138 (62·4)
3	62 (28·1)
**Ki-67 pre-NACT, %**	
median (IQR)	55 (35-70)
Mean (SD)	53·3 (22)
**Ki-67 post-NACT, %**	

median (IQR)	45 (15,70)
Mean (SD)	43·5 (28)
**Abbreviations:** N, number; NACT, neoadjuvant chemotherapy; IQR, interquartile range; SD, standard deviation; T, tumour.	

Table S9. Comparative analysis for Ki-67 metrics and different pairs of measures of Ki-67, using Harrell C-statistic (C-index) and time-dependent ROC curves (Area Under the Receiver Operating Characteristic curve, AUROC).

**Measure of Ki-67 among the same patients with residual disease** (continuous variable)	**Univariable Cox regression[1] (without additional covariates)**			**Multivariable Cox regression (including covariates[2])**		
	Harrell C-statistic	*5-year AUROC*	*10-year AUROC*	*Harrell C-statistic*	*5-year AUROC*	*10-year AUROC*
**Without any Ki-67**	-	-	-	0·790	0·76	0·70
**Pre-NACT Ki-67**	0·648 (0·598-0·694)[3]	0·59	0·53	0·795	0·76	0·70
**Post-NACT Ki-67**	0·721 (0·673-0·765)	0·69	0·63	0·810	0·77	0·73
**Absolute change (AC) of Ki-67**	0·674 (0·626-0·723)	0·65	0·63	0·797	0·75	0·70
**Relative change (RC) of Ki-67**	0·686 (0·642-0·730)	0·66	0·63	0·801	0·77	0·71
**Pre-NACT Ki-67 + post-NACT Ki-67**	0·714 (0·671-0·768)	0·69	0·64	0·809	0·77	0·73
**Pre-NACT Ki-67 + RC of Ki-67**	0·725 (0·685-0·771)	0·68	0·61	0·808	0·77	0·72
**Post-NACT Ki-67 + RC of Ki-67**	0·719 (0·672-0·766)	0·69	0·63	0·809	0·78	0·73

[1] Univariable Cox regression included only single or combination of measures of Ki-67
[2] Multivariable Cox regression models included covariates such as type of chemotherapy, age at diagnosis, clinical tumour size and node status at diagnosis, ER-status, PR-status, HER2-status, histological grade, and diagnosis year
[3] The 95% confidence interval was estimated using the bootstrap method with 1000 resamples.
Abbreviations: NACT, neoadjuvant chemotherapy; AC, absolute change of Ki-67; RC, relative change of Ki-67; AUROC, Area Under the Receiver Operating Characteristic curve.

Table S10. Comparison of relative change of Ki-67 versus post-NACT Ki-67 versus absolute change of Ki-67 for classifying patients with triple negative breast cancer (TNBC) and residual disease (RD), using the defined optimal cut-off values, reporting both crude and multivariable-adjusted hazard ratios (HRs).

**Risk group** (Patients with TNBC and RD)	**Category by relative change (RC) of Ki-67**			**Category by post-NACT Ki-67**			**Category by absolute change (AC) of Ki-67**		
	**Category**	**cHR (95% CI)**	**aHR (95% CI)[1]**	**Category[2]**	**cHR (95% CI)**	**aHR (95% CI)[1]**	**Category[3]**	**cHR (95% CI)**	**aHR (95% CI)[1]**
**Low-risk**	RC ≤ -0·48 (n = 95)	Reference (1·0)	Reference (1·0)	post-NACT Ki-67 ≤ 39% (n = 109)	Reference (1·0)	Reference (1·0)	AC ≤ -15·5 (n=104)	Reference (1·0)	Reference (1·0)
**Intermediate-risk**	-0·48 < RC ≤ 0·35	**2·42 (1·34–4·36)**	**2·61 (1·33–5·13)**	39% < post-NACT Ki-67 ≤ 66%	1·77 (0·90–3·45)	1·30 (0·60–2·82)	-15·5< AC ≤17·4	**2·40 (1·34-4·29)**	**2·43 (1·26-4·65)**
**High-risk**	RC > 0·35	**5·30 (2·61–10·76)**	**4·35 (1·94–9·77)**	post-NACT Ki-67 > 66%	**2·44 (1·45–4·11)**	**2·50 (1·38–4·53)**	AC> 17·4	**5·13 (2·66-9·90)**	**4·09 (1·96-8·53)**

[1] Multivariable Cox regression models included covariates such as age, type of neoadjuvant chemotherapy, tumour size, nodal status, tumour grade, and calendar year.
[2-3] Two optimal cut-offs (39% and 66% for post-NACT Ki-67; -15·5 and 17·4 for absolute change of Ki-67) were determined in the same approaches as the ones for relative change of Ki-67.
Abbreviations: TNBC, triple negative breast cancer; RD, residual disease; RC, relative change of Ki-67; NACT; neoadjuvant chemotherapy; AC, absolute change of Ki-67; cHR, crude hazard ratio; aHR, adjusted hazard ratio; CI, confidence interval.

Figure S1. (a) Hazard ratios (HRs) for breast cancer-specific mortality rates in the full cohort (N=2494), comparing patients with different levels of pre-NACT Ki-67 using the median as the reference cut-off. (b–d) Subtype-specific results for ER+/HER2-, HER2+, and TNBC, respectively. Estimates were adjusted for type of chemotherapy, age at diagnosis, tumour size, nodal status, ER status, PR status, HER2 status, grade, and year of diagnosis.

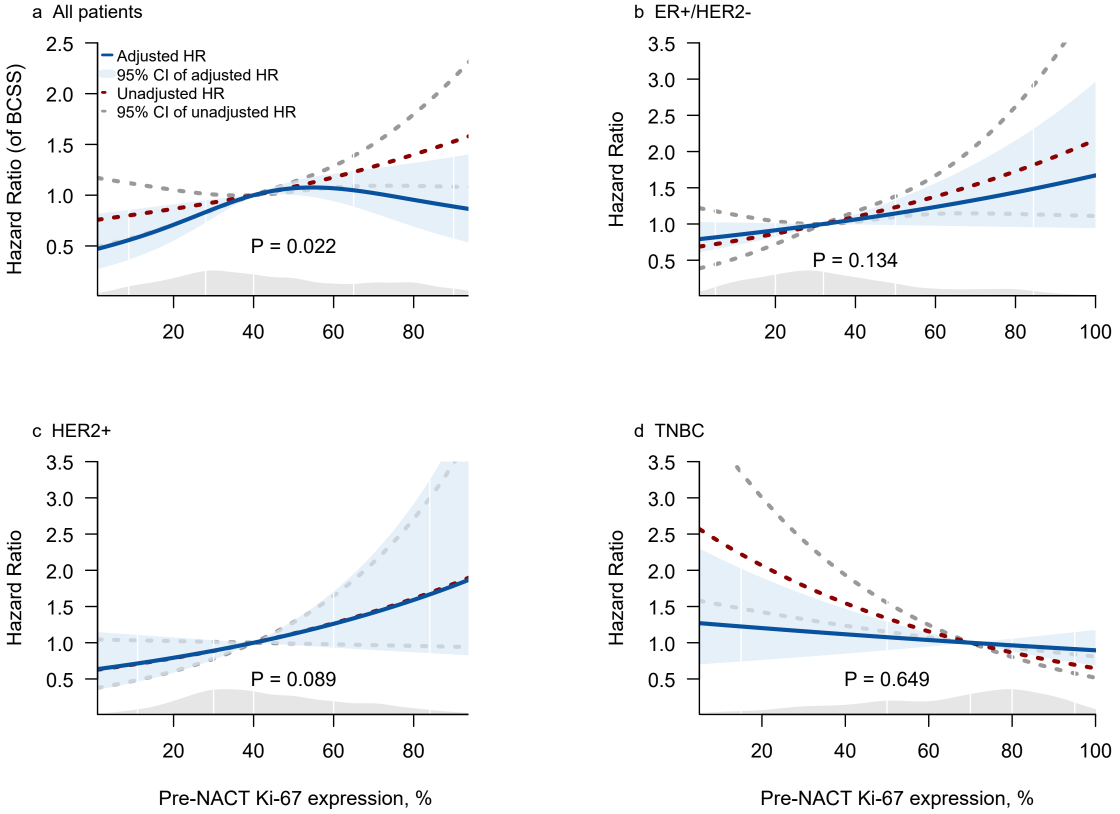

* The plots of hazard ratio were prepared using the R function Greg::plotHR(),[4] with Ki-67 being a continuous variable and modelled as restricted cubic splines in multivariable Cox regression analyses.[5] Time zero was the date of diagnosis of breast cancer. Adjusted hazard ratio was estimated through complete case analysis.

Abbreviations: NACT, neoadjuvant chemotherapy; ER, Estrogen receptor; HER2, Human epidermal growth factor receptor 2; TNBC, Triple negative breast cancer.

Figure S2. (a) Estimated crude and adjusted hazard ratios (for RFS events) comparing patients with different levels of pre-NACT Ki-67 to the median as a reference level, and corresponding 95% CIs, with adjustment for covariates such type of chemotherapy, age at diagnosis, tumour size, node status, ER-status, PR-status, HER2-status, grade, and diagnosis year. (b-d) corresponding results for three subtypes: ER+/HER2-, HER2+, and TNBC, respectively.

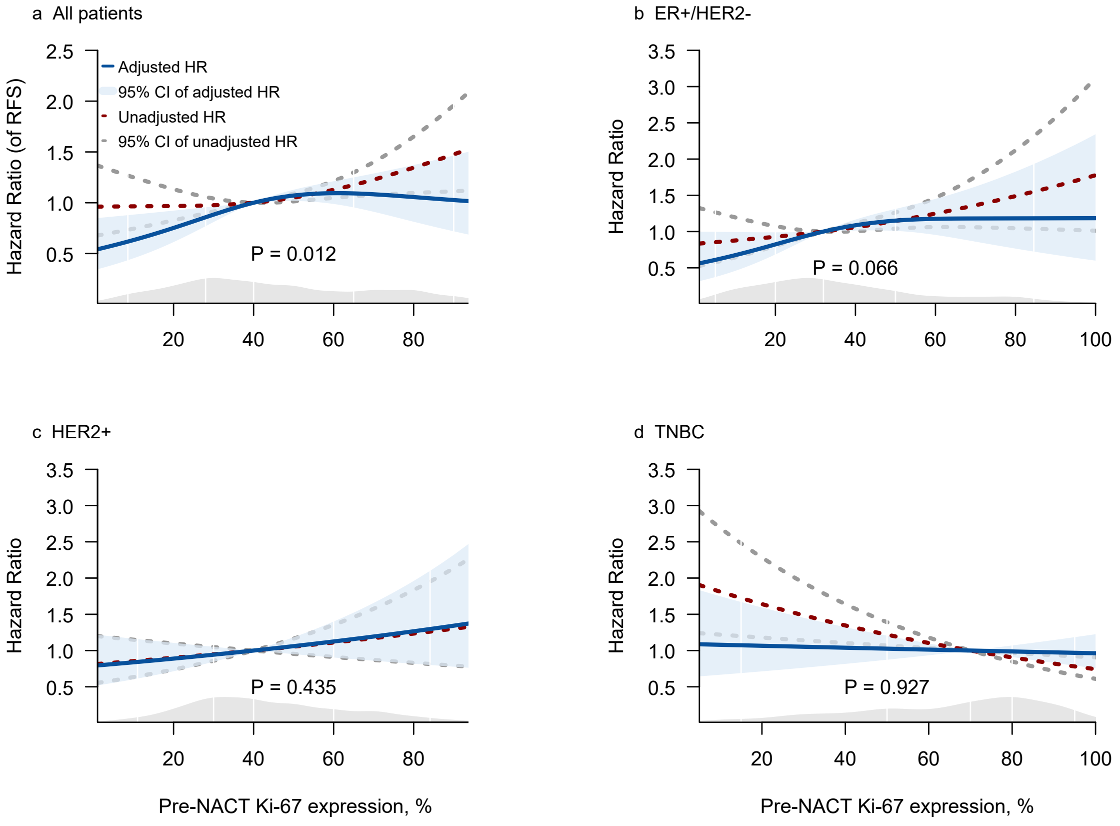

Abbreviations: RFS, recurrence-free survival; NACT, neoadjuvant chemotherapy; ER, estrogen receptor; PR, progesterone receptor; HER2, human epidermal growth factor receptor 2; TNBC, triple negative breast cancer.

Figure S3. Sensitivity analysis results from investigating whether estimated hazard ratio between continuous pre-NACT Ki-67 and breast cancer specific survival (BCSS) was robustness due to missing covariates (mainly compared to a in Figure S1). Multiple imputation methods[6] were used based on 500 imputed data sets.

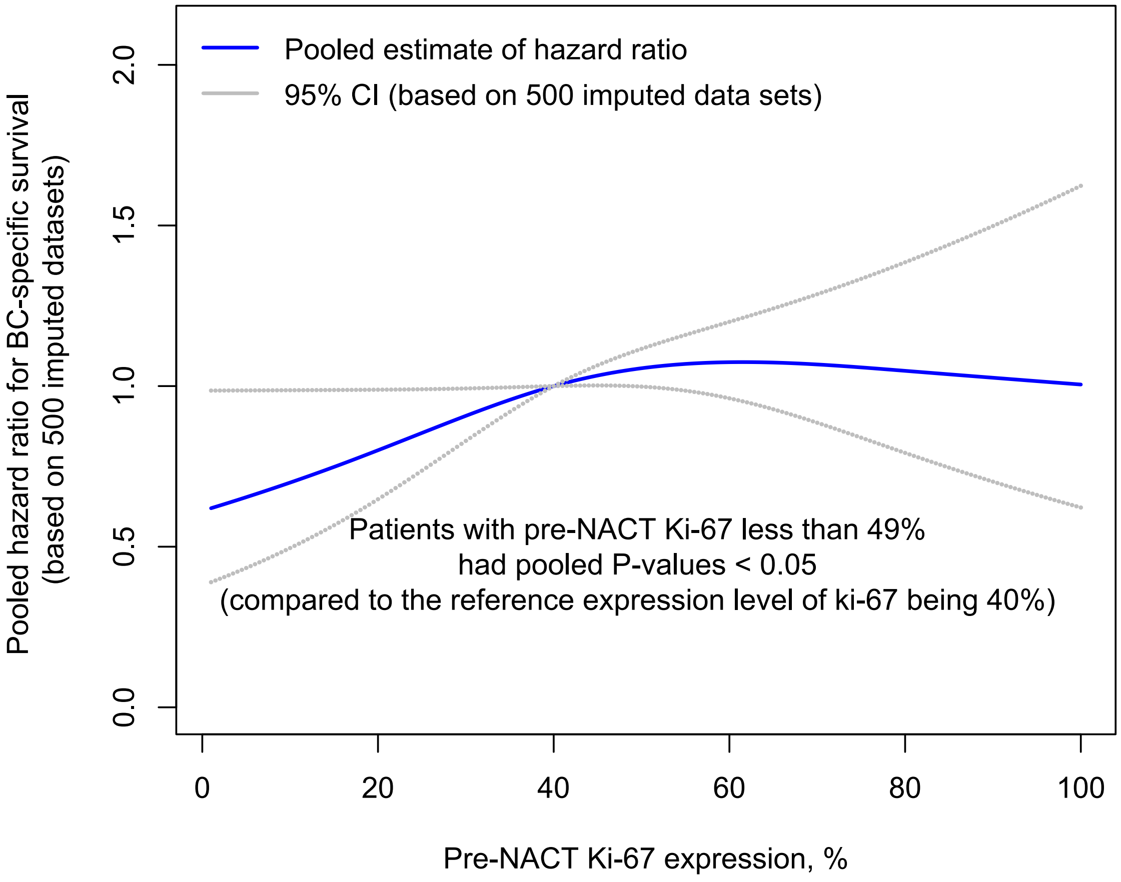

Abbreviations: NACT, neoadjuvant chemotherapy.

Figure S4. Unsupervised methods (Gaussian Mixture Models) that were applied to a. Patients with ER+/HER2- BC and residual disease and b. Patients with TNBC and residual disease to investigate whether pre- or post-NACT Ki-67 predominates in defining two clusters.

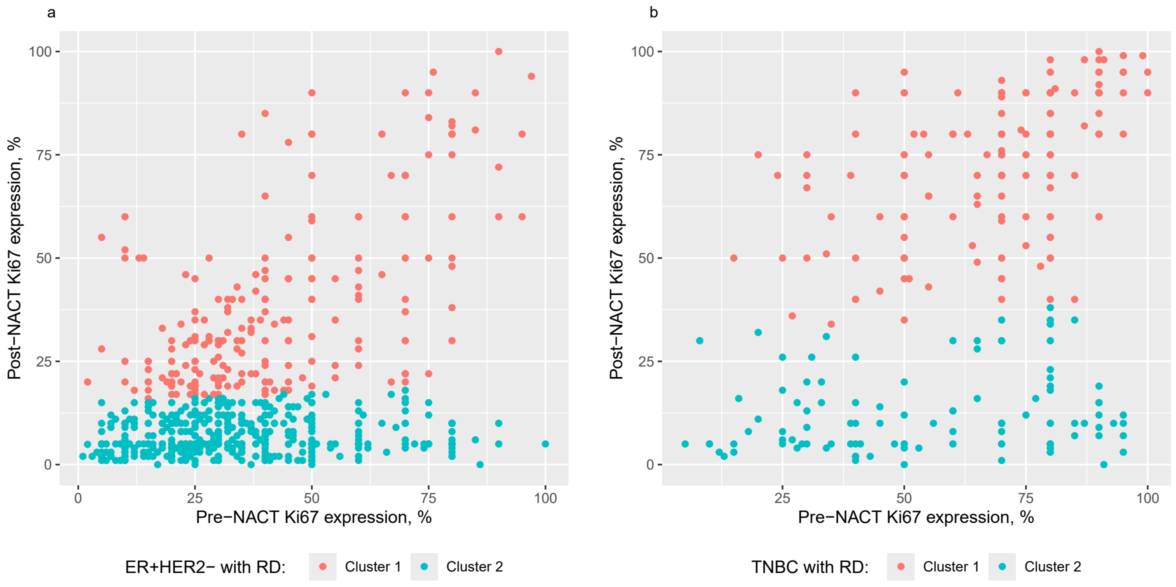

Abbreviations: NACT, neoadjuvant chemotherapy; BC, breast cancer; ER, Estrogen receptor; HER2, Human epidermal growth factor receptor; TNBC, Triple negative breast cancer; GMM, Gaussian Mixture Models.

Figure S5. Sensitivity analysis results from investigating whether estimated hazard ratio between continuous post-NACT Ki-67 and breast cancer specific survival (BCSS) was robustness due to missing covariates (mainly compared to a in Figure 3). Multiple imputation methods[6] were used based on 500 imputed data sets.

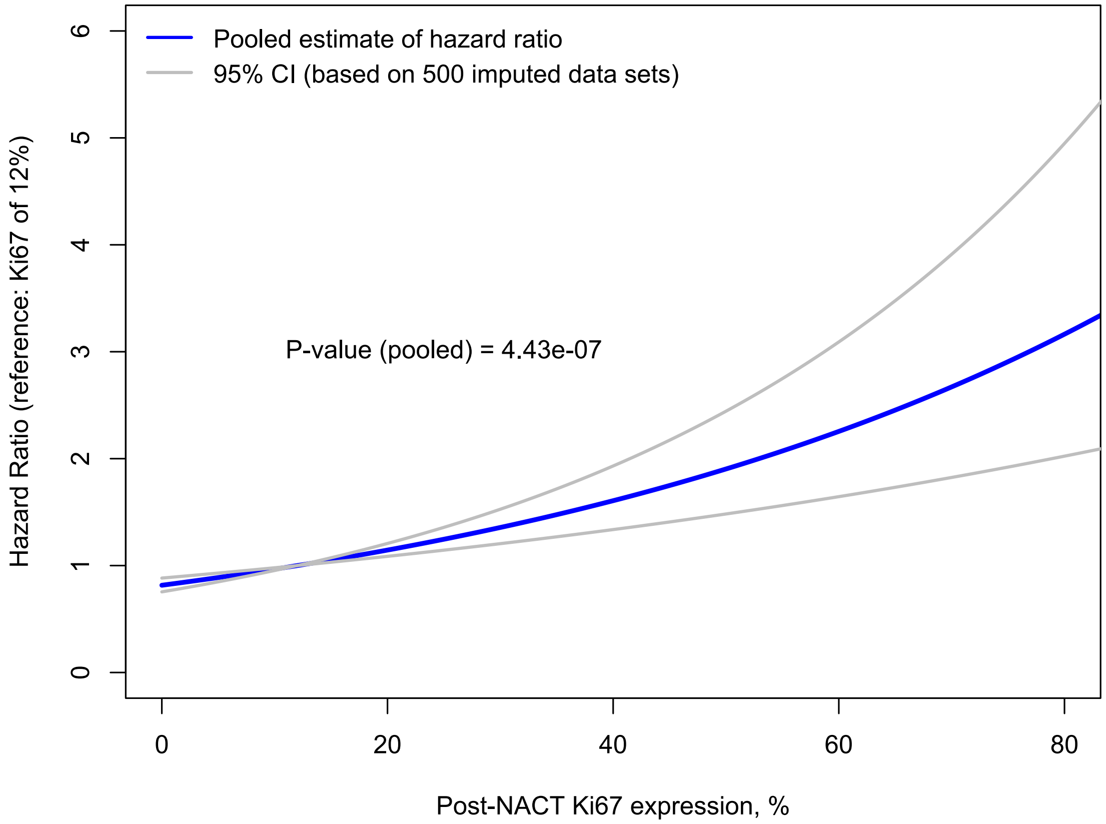

Abbreviations: NACT, neoadjuvant chemotherapy.

Figure S6. (a) Estimated hazard ratios (for RFS events) comparing patients with different levels of post-NACT Ki-67 to the median as a reference level, and corresponding 95% CIs, with adjustment for covariates such type of chemotherapy, age at diagnosis, tumour size, node status, ER-status, PR-status, HER2-status, grade, and diagnosis year. (b-d) presented corresponding results for three subtypes: ER+/HER2-, HER2+, and TNBC, respectively.

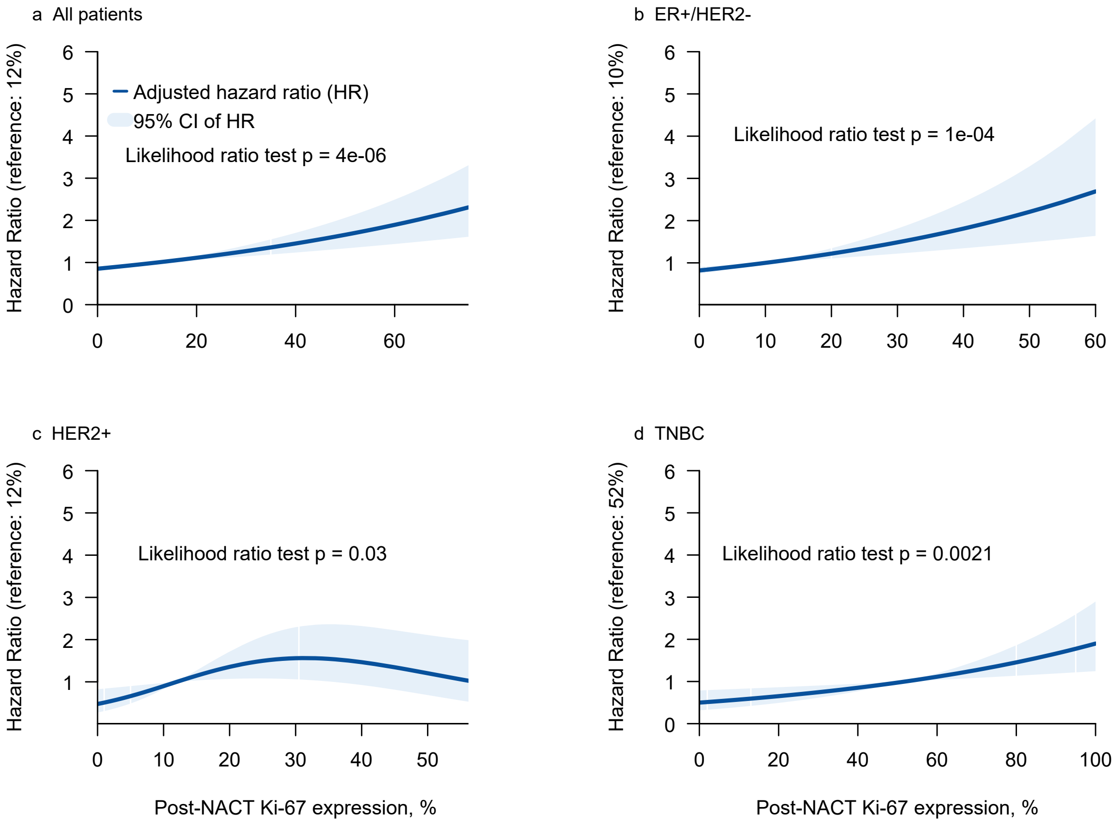

Abbreviations: RFS, recurrence-free survival; NACT, neoadjuvant chemotherapy; ER, estrogen receptor; PR, progesterone receptor; HER2, human epidermal growth factor receptor 2; TNBC, triple negative breast cancer.

Figure S7. Association between Neo-Bioscore and breast cancer specific survival (BCSS) among 1734 patients who had evaluable Neo-Bioscore (a) and available subgroups by: (b) ER+/HER2- , (c) HER2+, and (d) TNBC, respectively.

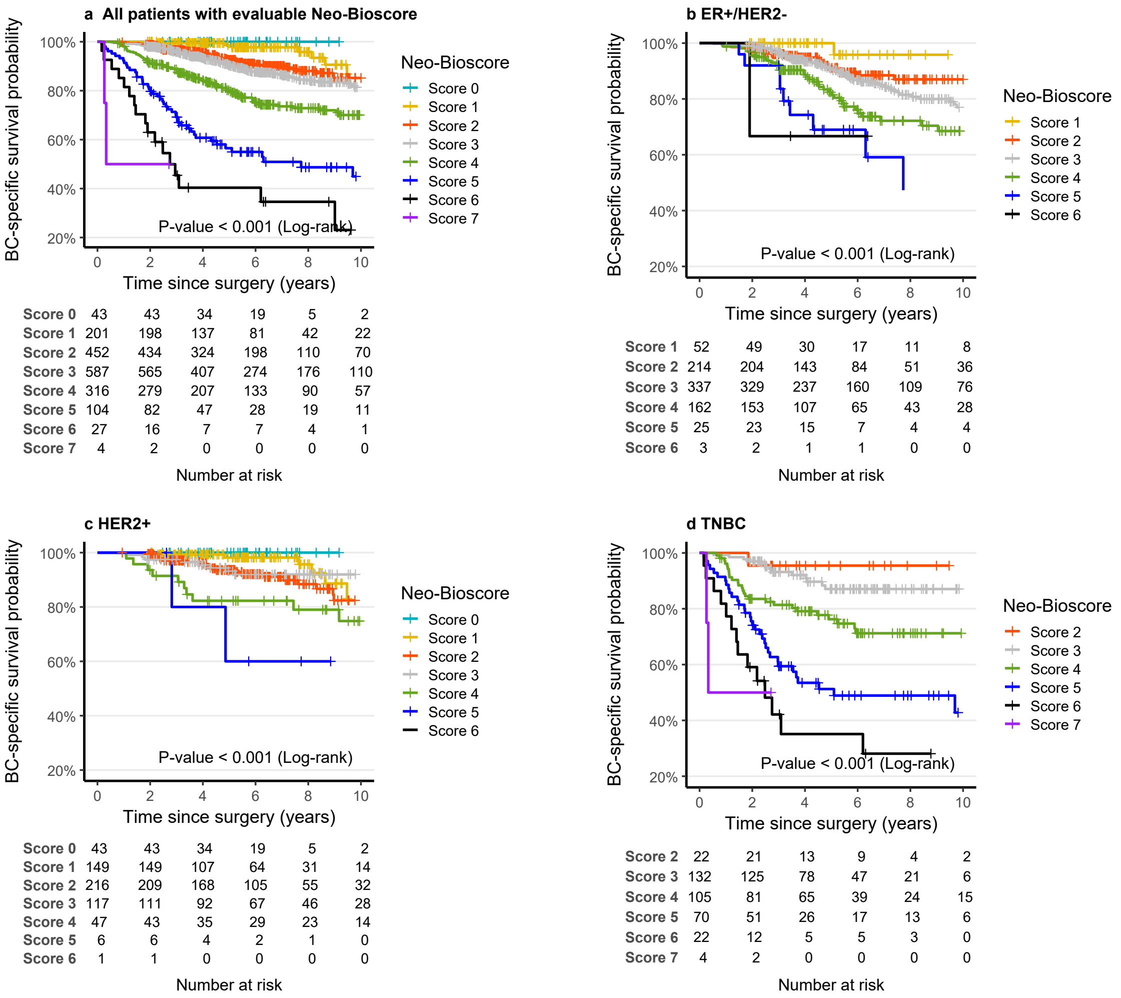

Abbreviations: NACT, neoadjuvant chemotherapy; ER, Estrogen receptor; HER2, Human epidermal growth factor receptor 2; TNBC, Triple negative breast cancer.

Figure S8. Association between Neo-Bioscore and breast cancer specific survival (BCSS) among 1085 patients with residual disease, having evaluable Neo-Bioscore, as well pre- and post-NACT Ki-67.

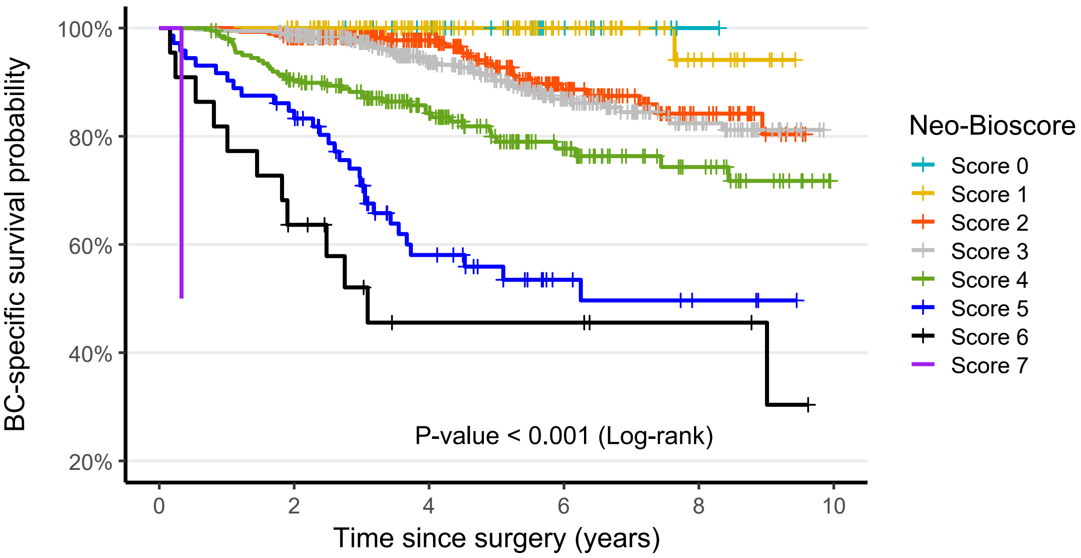

	0	2	4	6	8	10
**Score 0**	28	28	20	9	2	1
**Score 1**	111	108	64	30	12	2
**Score 2**	281	266	180	85	36	15
**Score 3**	368	354	225	128	71	39
**Score 4**	201	172	116	60	34	12
**Score 5**	72	60	30	15	11	8
**Score 6**	22	13	6	6	4	1
**Score 7**	2	1	0	0	0	0

Number at risk

Abbreviations: BC, breast cancer; NACT, neoadjuvant chemotherapy.

Figure S9. Using Cox regression models on 1085 patients with evaluable Neo-Bioscore and accessible pre- and post-NACT Ki-67, we evaluated prognostic performance of Neo-Bioscore with addition of continuous pre-NACT Ki-67 (pre), post-NACT Ki-67 (post), or categorized relative change of Ki-67 (RC) by both (a) AIC values and (b) Concordance index (c-index), respectively.

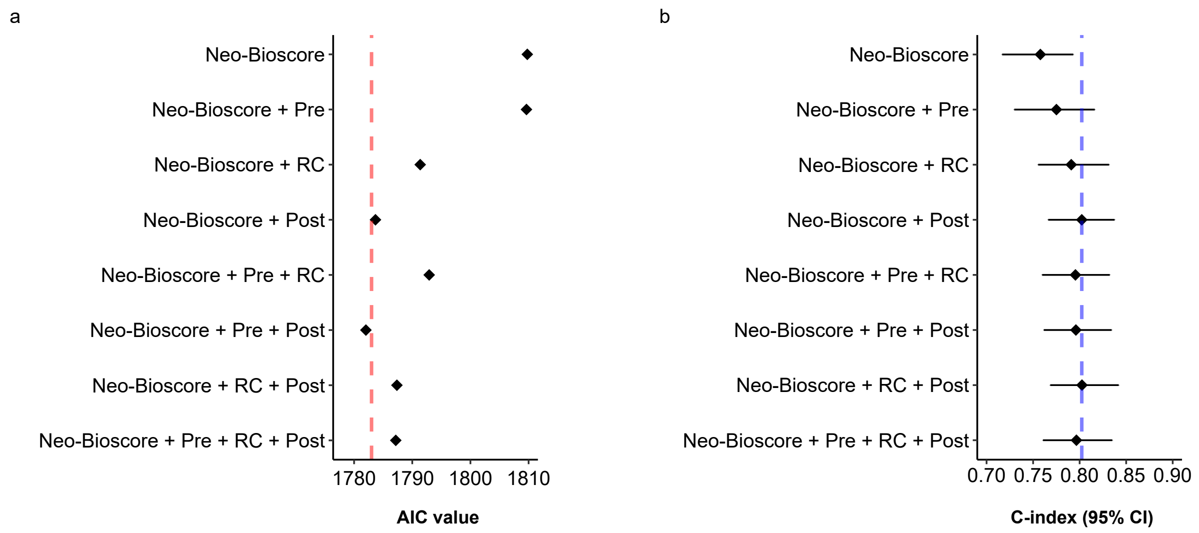

Abbreviations: AIC, Akaike information criterion; RC, Relative change; NACT, neoadjuvant chemotherapy.

Figure S10. (a) Estimated hazard ratios (for BCSS events) comparing patients with different levels of relative change of Ki-67 to the median as a reference level, and corresponding 95% CIs, with adjustment for covariates such type of chemotherapy, age at diagnosis, tumour size, node status, ER-status, PR-status, HER2-status, grade, and diagnosis year. (b-d) presented corresponding results for three subtypes: ER+/HER2-, HER2+, and TNBC, respectively.

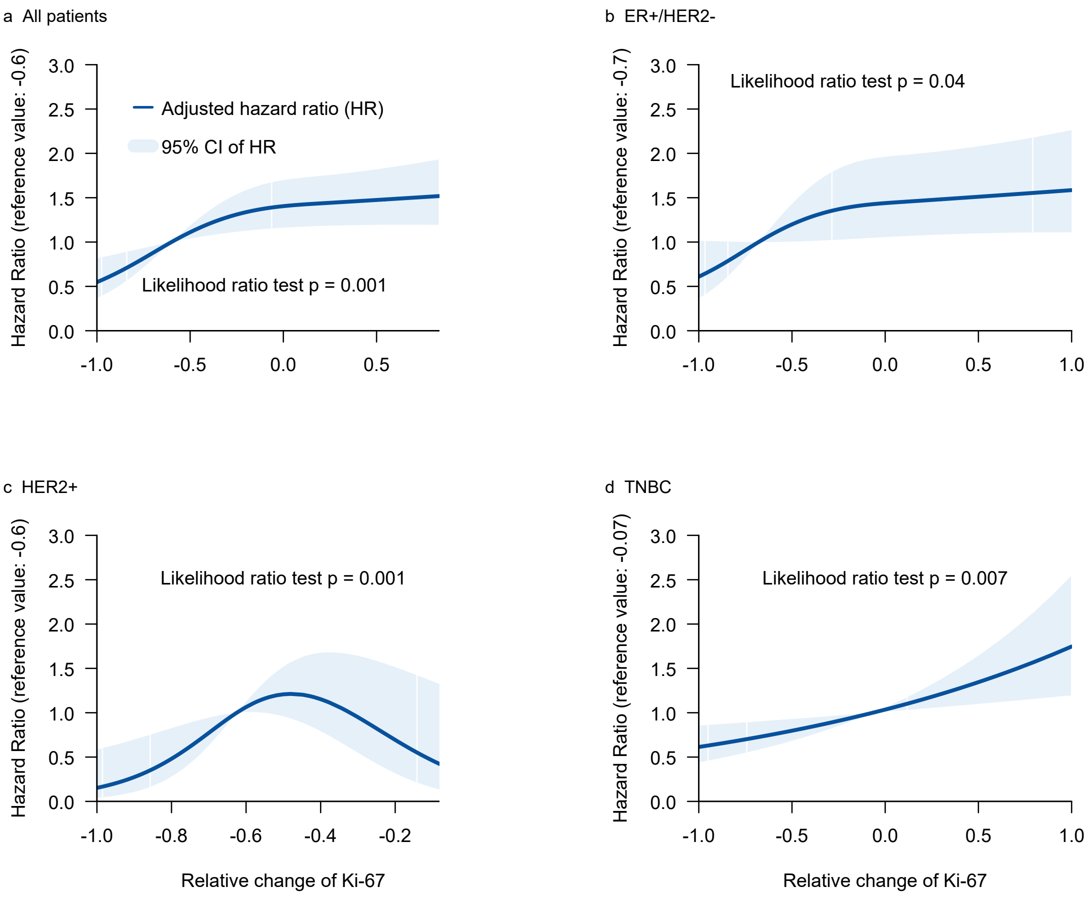

Abbreviations: BCSS, breast cancer specific survival; NACT, neoadjuvant chemotherapy; ER, estrogen receptor; PR, progesterone receptor; HER2, human epidermal growth factor receptor 2; TNBC, triple negative breast cancer.

Figure S11. (a) Estimated hazard ratios (for RFS events) comparing patients with different levels of relative change of Ki-67 to the median as a reference level, and corresponding 95% CIs, with adjustment for covariates such type of chemotherapy, age at diagnosis, tumour size, node status, ER-status, PR-status, HER2-status, grade, and diagnosis year. (b-d) presented corresponding results for three subtypes: ER+/HER2-, HER2+, and TNBC, respectively.

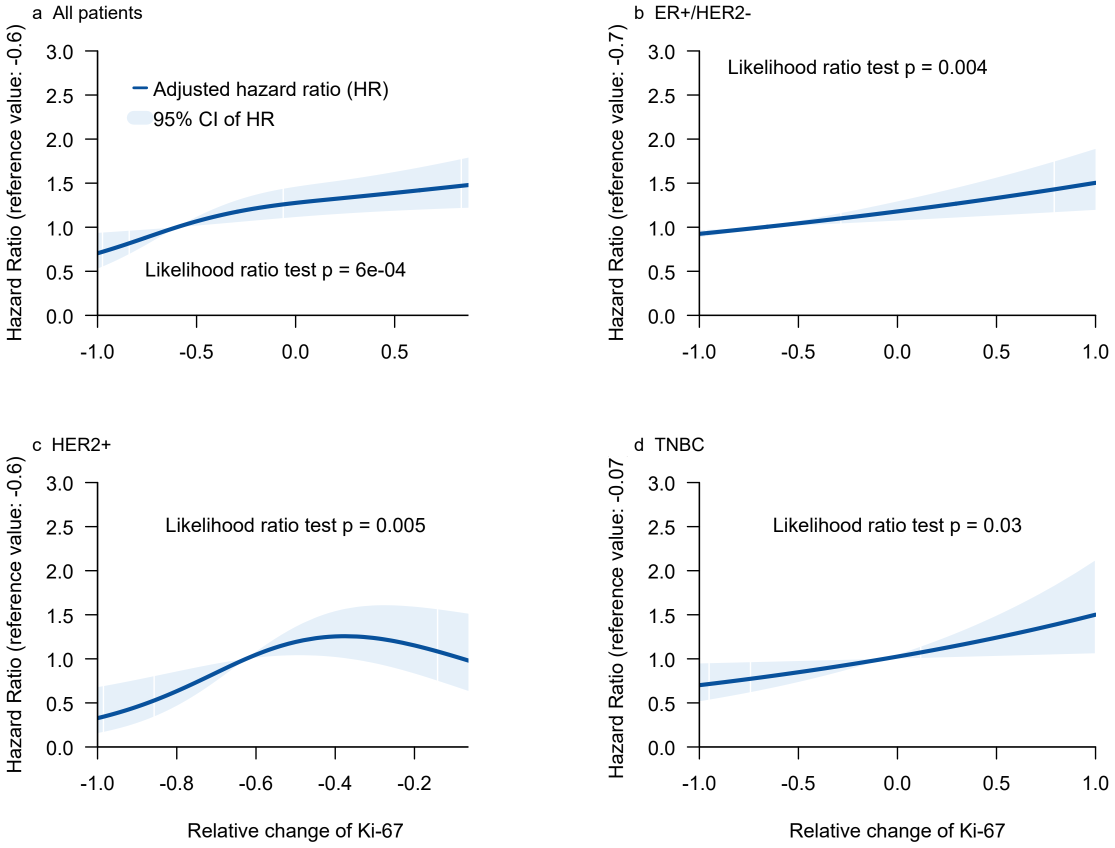

Abbreviations: RFS, recurrence-free survival; NACT, neoadjuvant chemotherapy; ER, estrogen receptor; PR, progesterone receptor; HER2, human epidermal growth factor receptor 2; TNBC, triple negative breast cancer.

Figure S12. Spearman correlation heatmap presenting associations of different Ki-67 metrics.

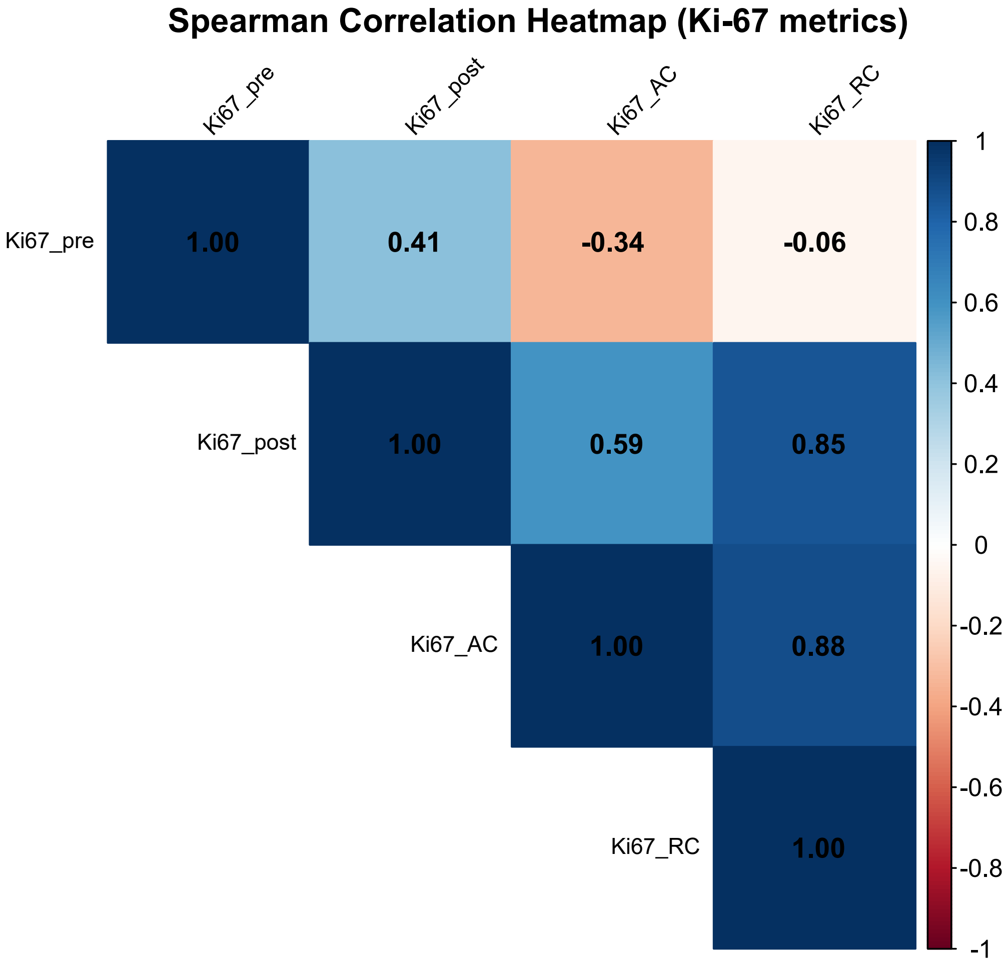

Abbreviations: AC, absolute change; RC, relative change.

References

1. Mittendorf EA, Vila J, Tucker SL et al. The Neo-Bioscore Update for Staging Breast Cancer Treated With Neoadjuvant Chemotherapy Incorporation of Prognostic Biologic Factors Into Staging After Treatment. *Jama Oncology* 2016;**2**:929–936.
2. Laas E, Labrosse J, Hamy AS et al. Determination of breast cancer prognosis after neoadjuvant chemotherapy: comparison of Residual Cancer Burden (RCB) and Neo-Bioscore. *Brit J Cancer* 2021;**124**:1421–1427.
3. Kantor O, Laws A, Pastorello RG et al. Comparison of Breast Cancer Staging Systems After Neoadjuvant Chemotherapy. *Annals of Surgical Oncology* 2021;**28**:7347–7355.
4. Gordon M, Seifert R. (2025). Greg: Regression Helper Functions. R package. 2.0.2. https://cran.r-project.org/package=Greg. April 2025.
5. Harrell FE. Regression modeling strategies: with applications to linear models, logistic regression, and survival analysis. New York: Springer; 2001.
6. Rubin DB. Multiple imputation after 18+ years. *J Am Stat Assoc* 1996;**91**:473–489.
